# Statistical modelling for precision agriculture: A case study in optimal environmental schedules for Agaricus Bisporus production via variable domain functional regression

**DOI:** 10.1371/journal.pone.0181921

**Published:** 2017-09-29

**Authors:** Efstathios Panayi, Gareth W. Peters, George Kyriakides

**Affiliations:** 1 Department of Statistical Sciences, University College London, London, United Kingdom; 2 Kyiakides Mushrooms Ltd., Larnaka, Cyprus; Tianjin University, CHINA

## Abstract

Quantifying the effects of environmental factors over the duration of the growing process on Agaricus Bisporus (button mushroom) yields has been difficult, as common functional data analysis approaches require fixed length functional data. The data available from commercial growers, however, is of variable duration, due to commercial considerations. We employ a recently proposed regression technique termed Variable-Domain Functional Regression in order to be able to accommodate these irregular-length datasets. In this way, we are able to quantify the contribution of covariates such as temperature, humidity and water spraying volumes across the growing process, and for different lengths of growing processes. Our results indicate that optimal oxygen and temperature levels vary across the growing cycle and we propose environmental schedules for these covariates to optimise overall yields.

## 1 Introduction

Modern agricultural production processes are evolving in a new era of sensor network technologies. These technologies are improving the monitoring of farming environments. The output of such technology has led to the rise of data analytics in this precision agriculture environment, in order to aid in the optimization of production and output of such farming practices.

Discussions on the evolution of farming processes in regard to the use of sensor network technologies are provided by [1], [2] and [3]. The majority of works in this area deal with questions related to large scale outdoor environments and aspects of sensor placement, sensor design, communications and power control. Few studies have begun to consider how to utilize all the sensor data being recorded, in order to perform statistical modelling that will inform and improve the process of farming crop management and optimization.

Furthermore, there is a new emerging field in this area which involves indoor commercial farming precision agriculture. In this field there is the a technological push for the adoption of specialized sensor networks that provide precision monitoring for optimization of commercial agriculture production.

There is now a growing amount of sensor network data available for a range of farming activities. However, there is a dearth of available statistical techniques, accessible by the farming industry to combine the data collected with understanding the farming process. This paper addresses this missing aspect by demonstrating how to perform statistically rigorous and accurate sensor data processing and then modelling of the sensor output with regard commercial considerations of yield and productivity. Following this, we also demonstrate how to utilize these models to then optimize commercial precision agricultural considerations. Examples of practical considerations that can be addressed by our proposed framework include: optimal sensory monitoring and control of key environmental conditions that will maximise yield and output of the precision agricultural farming environment.

In modern farming settings the data collected by sensor networks can be utilized to influence the farming practice and consequently may therefore be used for commercial results. Examples of such applications include increasing resulting product yields and reducing production costs and waste. There is thus a new challenge for statistical modelling to adapt methods of modelling and optimization to these new sensor network data outputs for utilization in the precision agriculture industry.

For instance, [4] utilized sensor network data to optimize irrigation processes in the farming cycle of cotton. In [5], the authors considered how sensor network data can be utilized to optimize greenhouse growing environments. These papers were focused on cost or efficiency gains in particular aspects of the production process. The sensor network studied in the first instance were specifically for moisture monitoring and in the second paper they also studied irrigation considerations in a green house growing environment. In our work, we develop a general statistical modelling approach for multiple sensor output types. We then use this framework to demonstrate how it may be applied to develop an optimization methodology that allows for modelling and environmental process control beyond just irrigation but applies, as we demonstrate to several precision growing conditions including temperature, humidity, irrigation, CO_2_ levels etc. This approach can be used to model and then optimize many desirable commercial considerations in precision agriculture. We chose to demonstrate this framework on yield maximization, however other considerations such as waste reduction, water reduction etc are equally applicable in our general modelling framework. Furthermore, we apply our framework for the optimization of production of a staple dietary ingredient for a number of countries, the protein rich mushroom.

The commercial cultivation of mushrooms has experienced explosive growth in the past 40 years. Total production has increased from 60,000 tons in 1978 to 25.7 million tons in 2011 to become a $24 billion industry [6]. It is particularly common in the Chinese diet, who were the first to cultivate the Lentinula edodes species in the 13th century [7]. Worldwide per-capita consumption has also increased four-fold between 1997 and 2012 [8].

In terms of global production, China currently accounts for around 70%, while in Europe, Poland is the biggest producer with almost 300,000 tons [9], followed by the Netherlands. Current cultivation methods can achieve an estimated yield of approximately 30 kilos per square meter, see details at http://www.mushroomidea.co.uk/about-mushrooms/facts-and-figures/index.cfm?articles_id=F1AE27D3-4AA4-4373-B699-E4CDEDE89024.

Three main genera constitute approximately 75% of the world’s mushroom supply [8]. Agaricus Bisporus (button mushroom), constitutes about 30%, while Pleurotus (oyster mushroom) accounts for approximately 27%. Lentinula edodes (shiitake mushroom) contributes another 17%. We study the cultivation process for the Agaricus Bisporus genus in this paper, which is by far the leading genus in Europe.

While there has been extensive research with regard to the preparation of the growth substrate for cultivation and its effect on the yield (see, e.g. [10] and citations within), there has been relatively little research on trying to identify the optimal environmental conditions for the growing process. Commercial cultivation is carried out in controlled conditions with set schedules for, e.g., temperature, humidity, oxygen, CO_2_ and water irrigation types and schedules. The preferred farming schedules for these different environmental controls differ amongst growers due to personal experience, as well as commercial considerations, budgetary constraints, logistics scheduling, yields and qualities required and farming time horizons.

A significant challenge faced in precision agriculture commercial applications is to optimize growing conditions with regard to a given commercial objective, such as yield maximization. In the case of Agaricus Bisporus yields this involves finding a means by which one can relate the scalar yield responses, observed from each farming production cycle, to the complex, multi-dimensional time-series of environmental factors collected from the sensor networks throughout the cycle.

It is clear that having constant values for the different variables throughout the growing process will lead to suboptimal production results in terms of both yields and product quality, and one therefore has to quantify the contribution of the environmental covariates at each point in the process. In this paper our sole focus is on the yield of mushrooms produced, though future works will also consider influences on product quality achieved for a given yield.

To address this problem we propose a solution based on an interesting new area of statistical regression modelling. In particular, we consider a functional regression approach, see [11] for a textbook-level discussion of the wider area of functional data analysis, in general in such models one relates the scalar response to one or more functional covariates. In this case, the covariates would be the time-series of temperature, humidity, water etc., over the growing process. Such a regression would result in a smooth functional coefficient that indicates at each point in the process whether the covariate contributes positively or negatively to the yield.

However, there are technical restrictions which prevent us from following such an approach (discussed in Section 4), which are due to the fact that the growing processes on each production run vary in length. The variation in length occurs both due to commercial considerations to do with logistics, supply and demand considerations and also quality and quantity considerations that are related to grower’s preferences, such as when they observe that there is limited utility in furthering the growing process due to the current demand or quality of product produced in the particular growth cycle.

Addressing this issue of different length time-series of the observed covariates is important because growers target different target environmental schedules according to whether they are targeting a shorter growth period or a longer growth period. In addition, these schedules may be modified depending on the particular substrate/compost utilized. Therefore it is important to carefully incorporate this feature into the regression modelling approach, and certainly naive truncation or rounding may produce spurious results. In this paper, we therefore employ a regression technique termed Variable-Domain Functional Regression (VDFR), recently proposed by [12]. As the name implies, this enables us to use our functional data, where each time-series data point is of different length, without having to resort to any compression to obtain fixed-length data. Similar to standard functional regression, this results in a functional coefficient, but which now varies smoothly in 2 directions: Firstly, over the growing process, but also across the different possible lengths of the growing process.

Our results indicate that there is a very clear effect of oxygen levels on total yield. In particular, the model verifies that a lower oxygen level is found to be beneficial in the first two-thirds of the growing process, while higher oxygen levels are found to contribute to increasing yields in the final third. For temperature, higher temperatures are beneficial for the first half of the process, while in the second half these higher temperatures are only favourable for longer growing process durations.

### 1.1 Contribution and structure

Summarizing the main contributions of this paper we highlight the following key aspects of relevance to statistical modelling of output from sensor networks in precision agriculture settings:

- A functional regression based framework is developed for the statistical modelling of output from sensor network that produces multivariate time series data and linking this to the variation of outputs of the production process such as crop yield. This model framework is particularly focused on the setting of precision agriculture which poses a novel challenge for such functional regression frameworks, in that each repeated trial is of an unequal length. This variable length is due to variable harvest times resulting from commercial constraints on production.
- A variable domain functional regression framework is then developed to overcome this unique challenge in the precision agriculture setting and the model is applied to a detailed study of indoor precision agriculture in the area of mushroom production.
- Furthermore, we then demonstrate how to utilize the statistical model developed to optimize the production yield. This is possible to achieve since the model developed links the relationship between environmental conditions being monitored by the sensor network to a commercial variable of interest, in our case yield allowing us to obtain an optimal growing environmental control output. That is, we design a simple and effective model based optimal growing schedule for environmental variables that are monitored and can be controlled in a precision indoor growing environment. These variables are defined in detail in Tables 1, 2 and 3 and included: compost type; total yield in kg per square meter of growing surface; pH level of the soil; moisture content of the soil; are of growing surface; weight of compost per square meter; duration of growing period in number of days; air temperature in degree Celsius; percentage of oxygen context in growing environment; air conditioning capacity; CO_2_ produced in g per hour, per square meter of compost; amount of water evaporated in g per hour per square meter of compost.

**Table 1:** Compost providers and irrigation systems for the 92 growing periods under consideration.

	Irrigation system	
Compost provider	New	Old
**A**	68	0
**B**	0	24

**Table 2:** Scalar variables used in our models.

Variable	Description
**YieldTotalkgsqm**	The total yield (in kg) per squared metre of growing surface. This is the response variable.
**pH**	This is the pH of the compost, provided by the compost provider. The industrial process used to determine this is similar to that described by [10].
**MC**	This is the moisture content of the compost, i.e. the percentage of water that constitutes it.
**Growingareasqm**	The area of the growing surface.
**Compostfilledkgsqm**	The weight of compost per squared metre of area.
**totdays**	The total duration of the growing period (in number of days).

**Table 3:** Functional variables used in our models.

Variable	Description
**AirtemperatureC**	The temperature in degrees Celsius as measured by a termperature sensor in the middle of the growing room.
**Oxygen**	The percentage of oxygen in the growing room air.
**HumDeficitinletgkg**	The capacity of the air coming in to the growing room (in g/kg) to absorb excess moisture evaporating from the growing beds, independently of the air temperature.
**Co2productionghm2**	The amount of CO_2_ produced (in g) per hour, per squared metre of compost.
**Evaporationghm2**	The amount of water that has evaporated (in g), per hour, per squared metre of compost.

The rest of this paper is structured as follows: Section 2 provides a short background regarding mushroom production and reviews studies related to yield modelling in this area. In Section 4 we review the area of functional data analysis, focusing on functional regression, and introduce VDFR. Section 5 outlines the VDFR results, along with a detailed interpretation. Section 6 utilises these results within a Lagrange multiplier framework to obtain optimal environmental schedules for the grower. Section 7 concludes.

## 2 Mushroom production and yield modelling background

In this section we provide a brief overview of mushroom production. This is important to understand, in order to develop a practically meaningful framework for the modelling to be undertaken. Such context can aid in the understanding and selection of which environmental variables and processes are likely to affect the yield produced in the growing process. This in turn ought to be considered in the modelling process.

### 2.1 Basics of mushroom production

Mushrooms grow on vegetable waste, breaking down the organic material present. In commercial cultivation, various vegetable wastes can be used in the preparation of mushroom growth substrates. For instance, mushrooms can be grown on wheat straw, rice straw, sugar cane waste, coffee pulp and cottonseed hulls, among others. Typically the choice of growth medium will depend on the type of mushroom selected by the grower and the availability and abundance of such raw materials.

The commercial cultivation of the edible mushroom Agaricus Bisporus, which is the focus of this paper, uses a fermented and pasteurised substrate as a growth medium. This usually consists of wheat straw, water, minerals and nitrogen sources, such as manure and urea and its preparation is usually broken down into three distinct phases.

**Phase I** is composting, which can be done either outdoors or indoors. Its main purpose is to release the nutrients present in the ingredients in such a way that they are easily accessible by the mushroom mycelium, once the latter is introduced. This is achieved by wetting the substrate, allowing it to self-heat and repeatedly turning it in order to mix and homogenize it. Through the microbiological and chemical processes involved in composting, the substrate is rendered selective by encouraging the growth of mushroom mycelium over other, competitive fungi.

Following composting, **phase II** ensues. The substrate is moved into purpose-built chambers, called tunnels, where the conditions of temperature, moisture content, oxygen levels and air flow are carefully controlled by specialised software and an array of sensors and environmental devices. Inside the tunnels, the substrate undergoes a pasteurisation process at high temperatures, where all undesirable organisms that can hinder mushroom development are destroyed. The compost is then gradually cooled down from around 58°C to 48°C over a period of 6-7 days in a process called conditioning. The purpose of conditioning is to release any excess ammonia compounds in the substrate, which cannot be tolerated by mushrooms and, also, to promote the growth of thermophilic microorganisms, like actinomycetes, which further the selectivity of the compost. This process is described in detail in the textbook-level discussions of [13, 14, 15] and analysed using electron microscopy by [16].

**Phase III** of substrate preparation involves the introduction of the mushroom mycelium by use of commercial spawn. Commercial spawn is a pure culture of mycelium, normally growing on cereal grains. It is very important to ensure that spawn is applied evenly in the compost to ensure an even colonisation of the substrate. Following spawn inoculation, the newly-introduced mycelium is allowed to fully colonise the substrate in a controlled environment over a period of 14-17 days. The substrate is then ready to be delivered to the commercial mushroom growers.

Mushrooms of the genus Agaricus Bisporus, however, cannot grow on compost alone. It has been found that the use of a protective, covering layer of casing soil placed over of the compost is imperative for growing mushrooms commercially [17]. Commercial growers and academic researchers alike have experimented with many types of materials to be used as casing soil for mushrooms, see e.g. [18, 19, 20, 21, 22].

In practice, a combination of peat and lime is used by most commercial growers of mushrooms, since peat has been found to have a great water-holding capacity [15]. This casing must be free of microorganisms that can become competitive to the mushroom mycelium.

Although we will not look into the casing soil in detail in this paper, it is important to point out its main functions as described in [23], which are:

- To assist in creating the appropriate microclimate for mushroom formation.
- To promote the development of fruit bodies through the provision of the necessary bacteria.
- To absorb large quantities of water and release that water gradually, so it acts as a water reservoir. Without the casing layer, it would be impossible to control the moisture content of the compost, as the surface would dry out by ventilation.
- Water transports nutrients from the core of the compost layer to the mushrooms on the top where it then evaporates. A wet casing soil with good structure will support this water movement and, thus, provide adequate nutrients for good mushroom growth.

### 2.2 Compost and environmental factor effects on yields

We should distinguish firstly between the two types of growth, i.e. mycelium and fruiting body growth. The mycelium is the vegetative part of the fungus, which branches into the soil, while the fruiting body is only produced after a period of sustained mycelium growth [24]. In general, optimal environmental conditions are more restrictive for fruiting body growth rather than for mycelium growth. As an example, [24] suggests that mycelial growth is found to occur over the range of 5 to 33°C, whereas fruiting occurs only from 13 to 24°C.

The variation in fruit body (mushroom) yields has been the subject of extensive research for approximately half a century, see e.g. [25]. With regards to the growing medium, yields are influenced by the composition of the substrate, but also casing, moisture content, CO_2_ etc. [10]. They found that a partial least squares model considering additionally ammonia, carbon, hydrogen, Cu etc was able to explain almost 90% of this variation. Because of heterogeneity in the raw material, as well as seasonal weather factors and nutrient availability, there will be a natural variation in the aforementioned characteristics [10] and thus in compost quality.

Variations in growing processes, as well as room conditions (e.g. temperature, humidity, evaporation, CO_2_ etc) will also be factors in the mushroom yield, but quantitative research in obtaining optimal environmental schedules for the duration of the growing process seems to be very limited. For Agaricus Bisporus, for example, [26] investigated the effect of manipulating the temperature schedule whilst keeping a constant CO_2_ level. They found that they were able to improve the synchronisation in the growth of mushrooms, resulting in fewer picking days, but with a contemporaneous decrease in yield.

We note here that the motivation for our analysis of environmental growing conditions is the study of methods that will increase the yield. Therefore, in this work we do not consider any potential impact on quality or shelf life. For research in this context, we mention briefly the work of [27], who has studied the effect of the time of harvest on post-harvest quality, while [28] suggested that factors such as compost composition, casing material, the irrigation process, environment and the flush number are likely to play a role. Research on Agaricus Bisporus shelf-life focuses mainly on the effect of post-harvest conditions, see e.g. [29] for a recent review of related work.

## 3 Experimental design and data

This observational study uses yield, compost and environmental factor data recorded by Kyriakides Mushrooms in their normal mode of commercial operation. Note that Kyriakides mushrooms is the largest grower in Cyprus, for information about their activities see http://www.kyriakides.com.cy/en/. In this section we outline the grower’s setup, providing details about the sensors used to monitor the growing process explaining how environmental data was collected for the subsequent studies we present.

Kyriakides Mushrooms operates a fully-automated system, which enables the grower a fine-grained control over the conditions throughout the growing process. The grower can set a predetermined schedule for the different environmental variables, but also intervene to target particular levels for certain periods of time. The system will then automatically determine the extent to which it should heat or cool air passing through the air duct, the amount of air being brought from outside (with the rest being recycled), as well as control fan speeds, so that target levels of e.g. air temperature, humidity and oxygen/CO_2_ are achieved.

**Fig 1:**
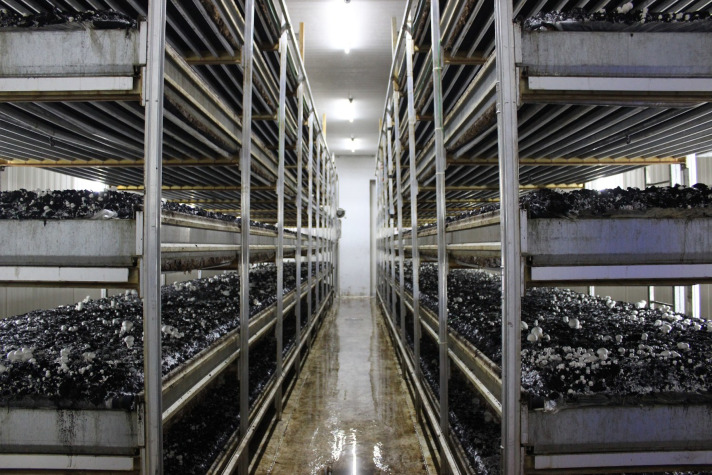
A photo of a typical growing room at Kyriakides Mushrooms.

Fig 1 shows a typical example of a growing room at Kyriakides Mushrooms during the growing process. It consists of 12 compost beds, with 4 probe-type PT100 4-wire compost temperature sensors, as well as further sensors for the room and air inlet temperatures. Temperature measurements from the probes is accurate to within 0.1°C. Relative humidity is measured using wet bulb and dry bulb air temperature probes, while CO_2_ is determined through specialised apparatus. Oxygen levels can then be calculated from the CO_2_ and relative humidity levels.

### 3.1 Data & descriptive statistics

Our data consists of 92 complete growing periods, the length of which varies between 35 and 43 days. For each growing period, we have a yield, as well as a number of scalar variables, which we describe in Table 2. In addition, we have observations for the environmental factors every half hour interval during the growing process in each trial. We will refer to such environmental factors as the ‘functional variables’, as this is how they will be treated in this analysis, and we present these variables in Table 3.

There are several other variables that could form part of our analysis, including compost temperature, evaporation, the position of the inlet and the speed of the fans, the temperature of the heating and cooling valves and others. Our choice regarding the variables is based on the environmental factors that are considered fundamental for the growing process, as well as a choice with regard to those variables that the grower has more control over through their own interventions.

For the purposes of standardisation among growing schedules used in different mushroom farms across the globe, in this paper we shall discuss the environment conditions in the growing room after the instant in time known to mushroom growers as ‘venting’: the introduction of ventilation (fresh air) into the room in order to halt the vegetative growth of mycelium and promote fruiting body development.

**Fig 2:**
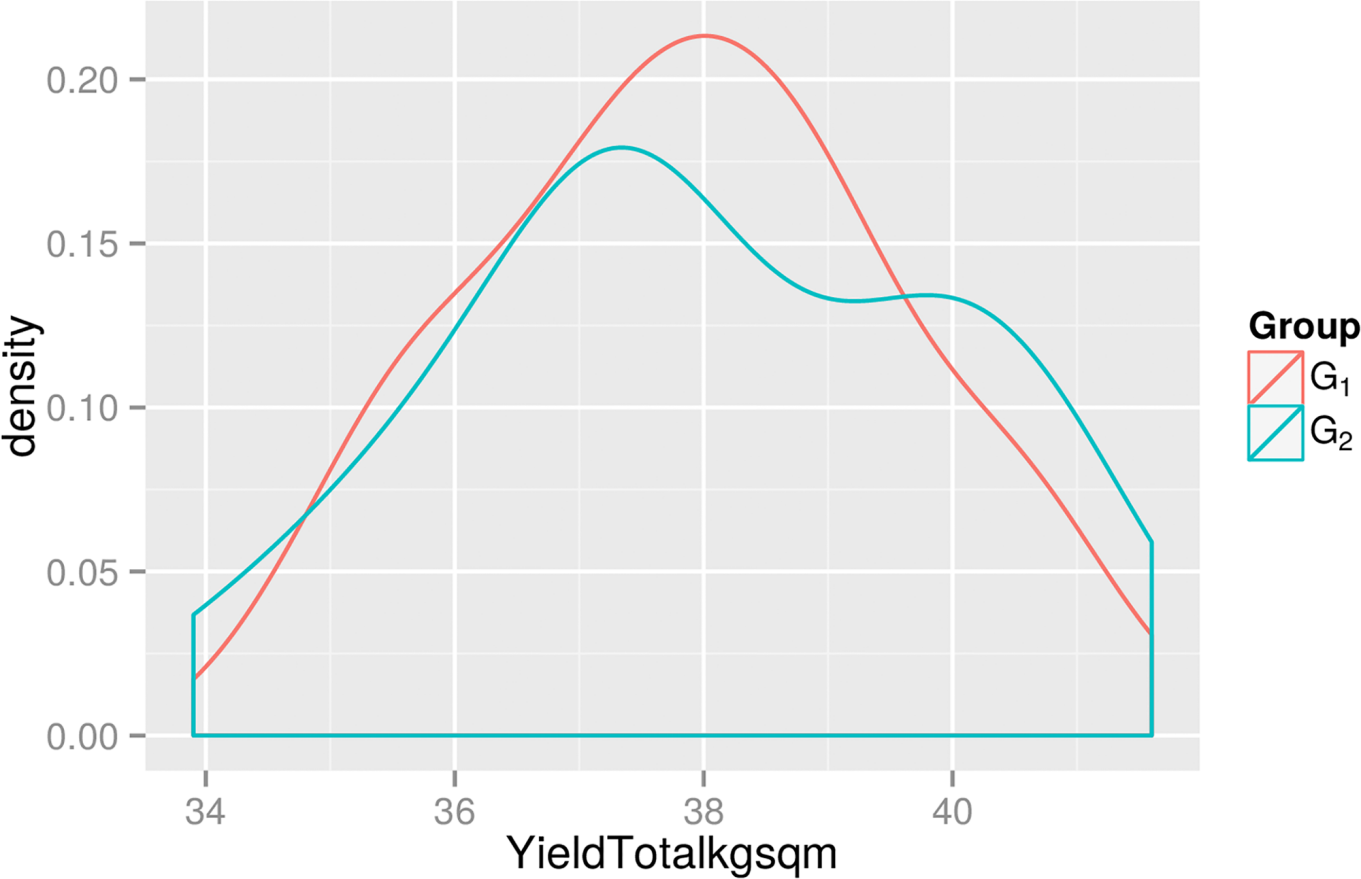
The smoothed density of the total yields in the two growing groups. The absolute values are obscured due to commercial considerations.

Within this dataset, there are 2 different compost providers (which we term ‘A’ and ‘B’) and 2 different irrigation systems (which we term ‘New’ and ‘Old’). However, all growing periods that use compost from provider A also use the New irrigation system, while growing periods that use compost from provider B use the Old irrigation system, and the breakdown is given in Table 1. Thus we term the first group (which uses provider A and the new irrigation system) as ‘G_1_’ and the second group as ‘G_2_’. Fig 2 shows the density for YieldTotalkgsqm—a two sample t-test provides no evidence at the 5% level for a difference in means.

## 4 Modelling approach

In deciding which modelling approach to adopt, one could draw upon a range of different time series based methods. For instance, one may consider works such as [30], [31] and [32] who developed various efficient ways to characterize complex systems via multi-scale time series analysis. In this manuscript we have selected a framework of modelling known as Functional Regression. This approach to modelling is particularly relevant to exploration of temporal causal relationships that will allow for precision control sequences to be obtained for optimization of commercial considerations such as yield of product produced.

### 4.1 Functional data analysis

In our model we focus on the effect of the environmental conditions throughout both the mycelium and fruiting body growth stages on the final yield. Because of the high dimensionality of the data (there are measurements every half hour for each of our environmental variables), there are advantages to representing this data in a functional form via Functional Data Analysis (FDA). Related techniques have already been used in various domains, from studying meteorological and kinesiology data [11] to financial market activity [33].

FDA has been an area of sustained academic interest for at least 25 years, with initial work by [34] and [35] giving rise to textbook-level discussions in [36] and [11]. Besides the representation of the functional data itself as a single functional object using splines, there have been innovations in obtaining functional regression modelling, e.g. for linear models see [11] and references within.

Compared to multivariate analysis, FDA can:

- Take into account the time-series structure of the data through smoothing [11]
- Represent the series of measurements parsimoniously as a single functional entity [37].

The following exposition of FDA is based on the work of [33]. FDA studies data which is represented as a function, where the domain of the function is usually time (as it is in the present case), although it could also be space or space and time. It enforces continuity in the time-series through smoothness constraints, and thus **z** = (*z*_1_, …, *z*_*n*_) are assumed to be observations from the functional *x*(*t*) at times **t** = (*t*_1_, …, *t*_*n*_) in the presence of noise:

$$\begin{array}{c} z=x(t)+\epsilon \end{array}$$ (1)

where ***ϵ*** is assumed to follow some parametric distribution. With this representation of *x*(*t*), one can then proceed to take derivatives of the function. Furthermore, such a representation allows one to also consider the use of multiple curves in a functional linear regression setting.

In this paper our interest is in the time-series of temperature, humidity, irrigation etc. To make concepts precise, let **y** denote a vector of temperature observations for a single yield. Our first goal is thus to represent this possibly noisy vector with a function *x*(**t**). At time *t*_*j*_, *j* = 1 … *n*, we will represent *x*(*t*_*j*_) using a basis expansion:

$$\begin{array}{c} x\left(t_{j}\right)=\sum_{k=1}^{K}\phi_{k}\left(t_{j}\right)c_{k} \end{array}$$ (2)

If we then have *N* functions then

$$x_{i}\left(t_{j}\right)=c_{i}^{T}\phi \left(t_{j}\right),i=1\ldots N.$$

We can see that for a sufficiently large *K*, one will be able to approximate a smooth function to any degree of accuracy. A common choice of spline in this setting is the B-spline as described in the following section 4.1.1 and section 4.1.2.

#### 4.1.1 Defining a basis system for functional data representation

Let **z** denote a vector of temperature observations for a single yield. Our first goal is thus to represent this possibly noisy vector with a function *x*(*t*). We will represent *x*(*t*) using a basis expansion: sufficiently large *K*:

$$\begin{array}{c} x\left(t\right)=\sum_{k=1}^{K}\phi_{k}\left(t\right)c_{k} \end{array}$$ (3)

If we then have *N* functions then

$$x_{i}\left(t\right)=c_{i}^{T}\phi \left(t\right),i=1\ldots N$$

We can see that for a sufficiently large *K*, one will be able to approximate a smooth function.

There are several example of bases which can be used, described in [33] and [11]. We will use a B-spline basis, which divides the observation range into sub-intervals, and then the basis functions are piecewise polynomials taking values in each sub-interval. One needs to define:

- The range [*t*_0_, *u*_*L*_] in which they take values.
- The order *m* of the spline, which is one higher than the highest degree polynomial. We consider cubic splines, i.e. *m* = 4, which is the most common setting.
- Break point and knot placement—these are the points which divide the observation range. We consider *L* − 1 interior knots, equally spaced over the range, and the interior knot sequence is denoted by **u** = (*t*_1_, …, *t*_*L*−1_). We select *L* = 16 in order to capture the features of the temperature curve over almost 2000 points.

Splines of increasing order are defined recursively:

$$\begin{array}{cc} B_{i,0}\left(u\right) & =\{\begin{array}{cc} 1 & \text{if}\;u_{i}\leq u<u_{i+1}\\ 0 & \text{elsewhere}. \end{array} \end{array}$$

$$B_{i,j+1}\left(u\right)=\alpha_{i,j+1}\left(u\right)B_{i,j}\left(u\right)+[1-\alpha_{i,j+1}]\left(u\right)B_{i+1,j}\left(u\right)$$

with ∑_*i*_ *B*_*i*,*j*_(*u*) = 1 and

$$\begin{array}{c} \alpha_{i,j}\left(u\right)=\{\begin{array}{cc} \frac{u-u_{i}}{u_{i+j}-u_{i}} & \text{if}\;u_{i+j}\neq u_{i}\\ 0 & \text{otherwise}. \end{array} \end{array}$$ (4)

**Fig 3:**
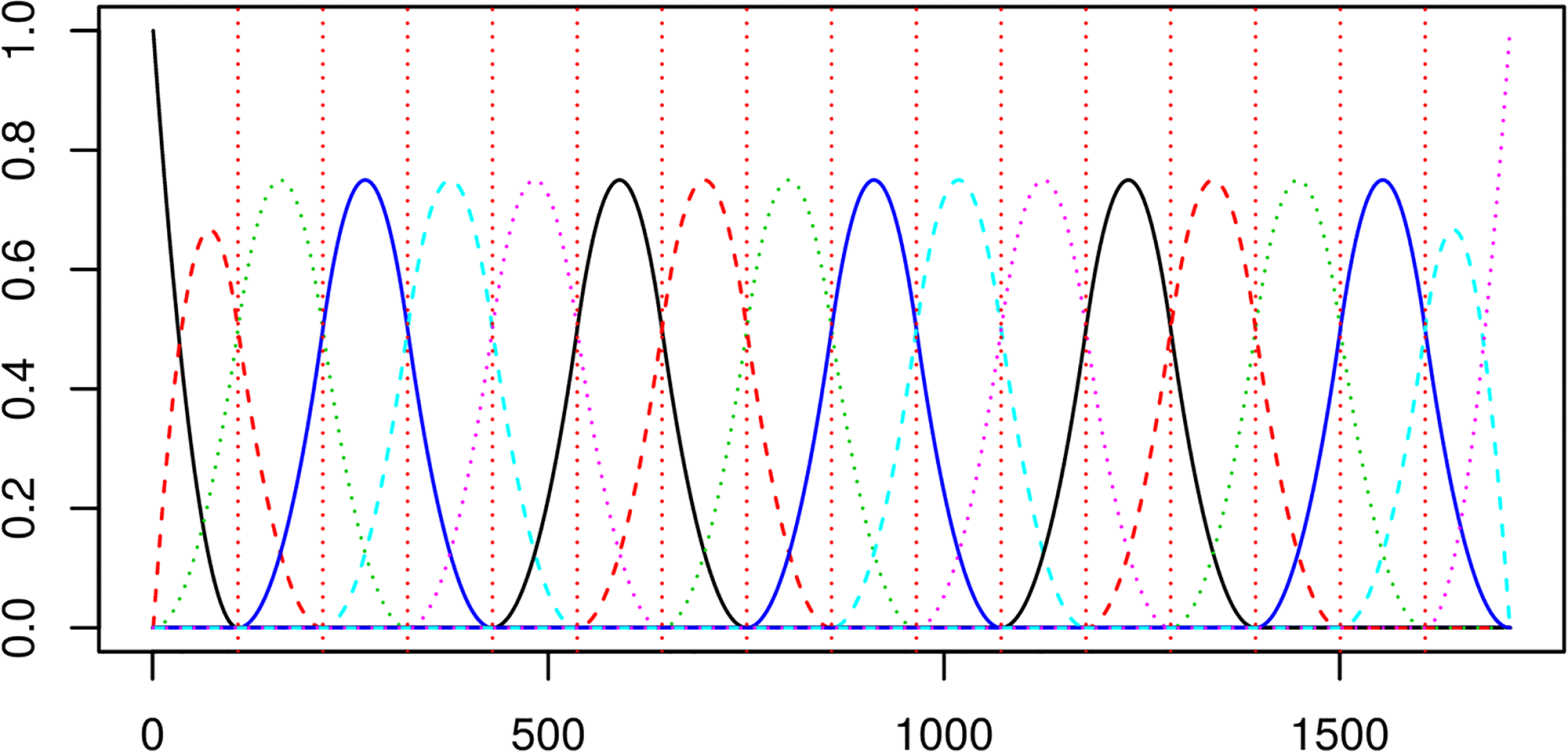
The basis functions used in our temperature curve representation. Note the x-axis reflects the time index for each 30min observation interval.

The spline function *S*(*u*) is then defined as

$$\begin{array}{c} S\left(u\right)=\sum_{k=1}^{m+L-1}c_{k}B_{k,m}\left(u\right) \end{array}$$ (5)

and illustrated in Fig 3.

B-splines have the compact support property, where each function can only be positive over at most *m* sub-intervals. The smoothness and continuity constraints are enforeced through matching the first two derivatives at the knot points.

**Fig 4:**
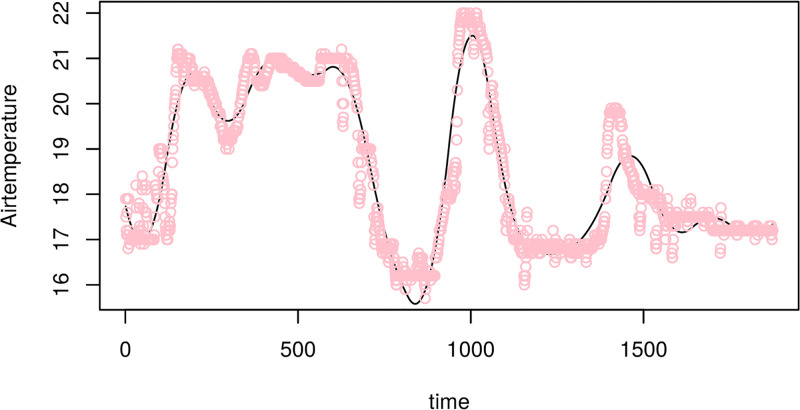
Cubic B-splines fit to the temperature time-series data (points) for a single growing period. Note the x-axis reflects the time index for each 30min observation interval.

This basis representation of the functional data is estimated using least squares. However, we note that it is important in practice to enforce smoothness of the functional representation obtained from the least squares solution. This is achieved by incorporating an additional constraint, known as a “roughness penalty”, which is added to the least squares criterion. Fig 4 shows an example of a cubic B-spline fit to the temperature time-series for a single growing period.

#### 4.1.2 Estimation of functional representations of covariate curves

The basis function representation of *x*(*t*) can be obtained through ordinary least squares, i.e. minimising

$$\begin{array}{c} SSE=\sum_{j=1}^{N}{\left(y_{j}-\sum_{k=1}^{K}c_{k}\phi_{k}\left(u_{j}\right)\right)}^{2}={\left(y-\Phi c\right)}^{\prime }\left(y-\Phi c\right) \end{array}$$ (6)

where Φ is an *N* by *K* matrix containing *ϕ*_*j*_(*t*_*k*_). From this we obtain

$$\begin{array}{c} \hat{c}={\left(\Phi^{T}\Phi \right)}^{-1}\Phi^{T}y \end{array}$$ (7)

and the vector of fitted values is

$$\begin{array}{c} \hat{y}=\Phi \hat{c}=\Phi {\left(\Phi^{T}\Phi \right)}^{-1}\Phi^{T}y \end{array}$$ (8)

from which we can see that Φ(Φ^*T*^Φ)^−1^Φ^*T*^ acts as a simple linear smoother. This approximation is only appropriate if we assume i.i.d errors, but this is not often the case with functional data. In order to enforce smoothness, we can add a roughness penalty to the least squares criterion

$$\begin{array}{c} PENSSE_{\lambda }={\left(y-\Phi c\right)}^{\prime }\left(y-\Phi c\right)+\lambda J\left(x\right) \end{array}$$ (9)

where λ is a tuning parameter and *J*(*x*) measures roughness, for example through the curvature *J*_2_(*x*) = ∫_*u*_[*D*^2^*x*(*u*)]^2^*du*, or, more generally, using any linear differential operator $J\left(x\right)=\int_{u}\sum_{k=1}^{m}\alpha_{k}D^{k}\left[\left(x\left(u\right)\right)\right]du$. The *D* operator is used to denote derivatives, such that *D*^0^*x*(*u*) = *x*(*u*) and $D^{m}x\left(u\right)=\frac{d^{m}x\left(u\right)}{du}$.

We impose the *J*_2_ roughness penalty in the estimation, as in areas where the function is highly variable, the square of the second derivative will be large. A theorem from [38] shows that when choosing *J*_2_(*x*) = ∫_*u*_[*D*^2^*x*(*u*)]^2^*du*, a cubic spline with knots at points *u*_*j*_ minimises *PENSSE*_λ_. Spline smoothing with the roughness penalty above is still a linear operation, where the smoother is now (Φ^*T*^Φ + λ**R**)^−1^Φ^*T*^, where

$$\begin{array}{c} R=\int D^{2}\phi^{\prime }\left(u\right)D^{2}\phi \left(u\right)du \end{array}$$ (10)

see [11] for a derivation. This is usually computed by numerical integration.

### 4.2 Functional linear models

Functional linear regression encompasses quite a large array of models, which can have:

1. A set of functional dependent variables and a scalar response, usually termed a functional linear model;
2. A set of scalar dependent variables and a functional response;
3. A set of functional dependent variables and a functional response. In this case, we have either a *concurrent model*, in which we assume that the response is only affected by the dependent variables at the same point of the domain of the functions, or a local influence model, which allows for integration over an interval of the domain [11].

[39] provides a recent review of the developments in this area, while [40] draws the three categories under a single framework. In modelling agricultural yields, we have a scalar response, therefore we will be focusing on the first category, i.e. functional linear models. For an example of the second category of functional regression models, see [11] and for the third, [33] provides an example for a concurrent model in a financial setting.

A general formulation of the functional linear model, where we have both functional and scalar covariates is

$$\begin{array}{c} y_{i}=\alpha_{0}+\sum_{k=1}^{q}\alpha_{k}z_{i,k}+\sum_{j=1}^{p}\int_{0}^{T}\beta_{j}\left(t\right)x_{i,j}\left(t\right)dt+\epsilon_{i} \end{array}$$ (11)

with $\epsilon_{i}∼N\left(0,\sigma^{2}\right)$.

The model is estimated using least squares, in a similar fashion to obtaining the functional representation above, using a further roughness penalty to ensure smoothness in the estimated covariate functions *β*_*j*_(*t*). This can also be extended to a generalised functional linear model, which again features a scalar output and functional covariates

$$\begin{array}{c} g\left(\mu_{i}\right)=\alpha_{0}+\sum_{k=1}^{q}\alpha_{k}z_{i,k}+\sum_{j=1}^{p}\int_{0}^{T}\beta_{j}\left(t\right)x_{i,j}\left(t\right)dt \end{array}$$ (12)

where $\mu_{i}=E[y_{i}]$ and *g*(⋅) is some link function. These types of models are covered by [41, 42].

### 4.3 Variable-domain functional regression

An issue with the standard functional regression approach described above is that it requires that the functional covariates are defined on a common interval, [0, *T*]. In our application described above, it is very common for the total length of the growth process to differ by a few days, due to many reasons, which include differences in the compost specification and activity, environmental conditions in the growing chambers as well as grower considerations (for example, the growing process is more likely to finish midweek to ensure that a new crop is prepared and set out for growing by Friday). It is obvious that the length of the growing process will have an effect on the yield, and we incorporate this consideration through a technique called variable-domain functional regression (VDFR), introduced by [12].

VDFR can now be introduced, which can be seen as an extension of Eq 11 that accounts for the variable domains:

$$\begin{array}{c} y_{i}=\alpha_{0}+\sum_{k=1}^{q}\alpha_{k}z_{i,k}+\sum_{j=1}^{p}\frac{1}{T_{i}}\int_{0}^{T_{i}}\beta_{j}\left(t,T_{i}\right)x_{i,j}\left(t\right)dt+\epsilon_{i} \end{array}$$ (13)

where $\epsilon_{i}∼N\left(0,\sigma^{2}\right)$. *T*_*i*_ is the length of the *i*-th growing process and the coefficient function is now bivariate. VDFR requires that the coefficient function *β*_*j*_(⋅) is smooth in both the *t* and *T* direction. Intuitively, this means that:

- For a single growing process, the effect of the coefficient function on weighting the covariate values will be similar within a short interval (say a couple of hours).
- If the growing process increases in length by a few hours, this should not have a large impact on the overall shape.

We are thus trying to obtain a smooth coefficient surface for *β*_*j*_(*t*, *T*_*i*_), for which we again employ splines, however, this time the splines are defined on a non-rectangular domain. Because of this, taking the generalization of B-splines to two dimensions will not be appropriate, as B-splines in 2d are defined over a rectangular surface. In addition, there a number of other issues relating to common spline bases, such as cubic splines and B-splines in this setting as discussed in [43] (p.152):

- The subjectivity in the choice of knot locations
- The bases are only useful for representing smooths of one predictor variable

Similar to [12], we thus use a thin plate regression spline basis (see [44] for an outline) for the domain of the coefficient function, which will be {*t*, *T*_*i*_: min_*i*_ *T*_*i*_ ≤ *t* ≤ *T*_*i*_ ≤ max_*i*_ *T*_*i*_}, i.e. a trapezoidal domain, since min_*i*_ *T*_*i*_ > 0. Thin plate regression splines are the equivalent of smoothing splines in the multidimensional setting. In the 2d setting, we want to find *f*(*x*_1_, *x*_2_) that minimizes

$$\begin{array}{c} -\Sigma_{i=1}^{n}l\left\{y_{i},f\left(x_{1},x_{2}\right)\right\}+\lambda \int \int {\left[\frac{\partial^{2}f}{\partial u^{2}}\right]}^{2}+2{\left[\frac{\partial^{2}f}{\partial u\partial v}\right]}^{2}+{\left[\frac{\partial^{2}f}{\partial v^{2}}\right]}^{2}dudv \end{array}$$ (14)

where *l* is some loss function. [45] and [46] showed that the unique minimizer to this expression is the natural thin plate spline with knots at the unique values of *x*_1_, *x*_2_. However, there are computational issues in employing this spline basis: for *n* data points it would require the estimation of *n* parameters, as well as an additional smoothing parameter, requiring O(*n*^3^) operations. [44] proposes approximations to thin plate splines which alleviate the computational obstacles to their use and removing the knot placement problem. The construction of these splines is quite involved, see [44] for details.

[47] showed the relationship between penalised splines and mixed models. In particular, they described how the ordinary nonparametric regression model

$$\begin{array}{c} y_{i}=f\left(x_{i}\right)+\epsilon_{i} \end{array}$$ (15)

could be estimated by penalised splines, and they showed that this estimate could be written as the best linear unbiased predictor of a mixed model

$$\begin{array}{c} y=Z\alpha +X\beta +\epsilon . \end{array}$$ (16)

where **Z*α*** denote the fixed component and **X*β*** denotes the random component, which can account for variability in one or more of the parameters.

Because of this connection, one can estimate penalized spline models (such as the VDFR model presented here) using mixed regression model estimation packages, such as the mgcv package in **R** [48]. In particular, the Generalized Additive Model (GAM) framework implemented in this package with the function gam will allow the response variable to depend on linear functions of smooth terms

$$\begin{array}{c} y_{i}=\ldots +\sum_{k=1}^{n}L_{ik}f\left(x_{i,k}\right)+\ldots \end{array}$$ (17)

where the *L*_*ij*_ are fixed weights. The right hand side of the expression above then can represent the weighted sum of the same smooth function evaluated at different covariate values. In this way, one can have the response depend on the integral of a smooth function, and one can see that this is appropriate for the estimation of Eq 13, which contains the expression $\int_{0}^{T_{i}}\beta_{j}\left(t,T_{i}\right)x_{i,j}\left(t\right)dt$. [12] provides an example for the estimation of a similar model, and the estimation in this paper is based on this example.

## 5 Method and analysis of results

In order to determine the relative importance of the scalar covariates listed in Table 2, we use them in a simple linear regression to determine their effect on the yield. Of these covariates, only Compostfilledkgsqm was found to be significant at the 5% level. In the interests of model parsimony, since we will have a potentially large number of spline parameters to estimate, further analysis considers only this scalar covariate.

**Fig 5:**
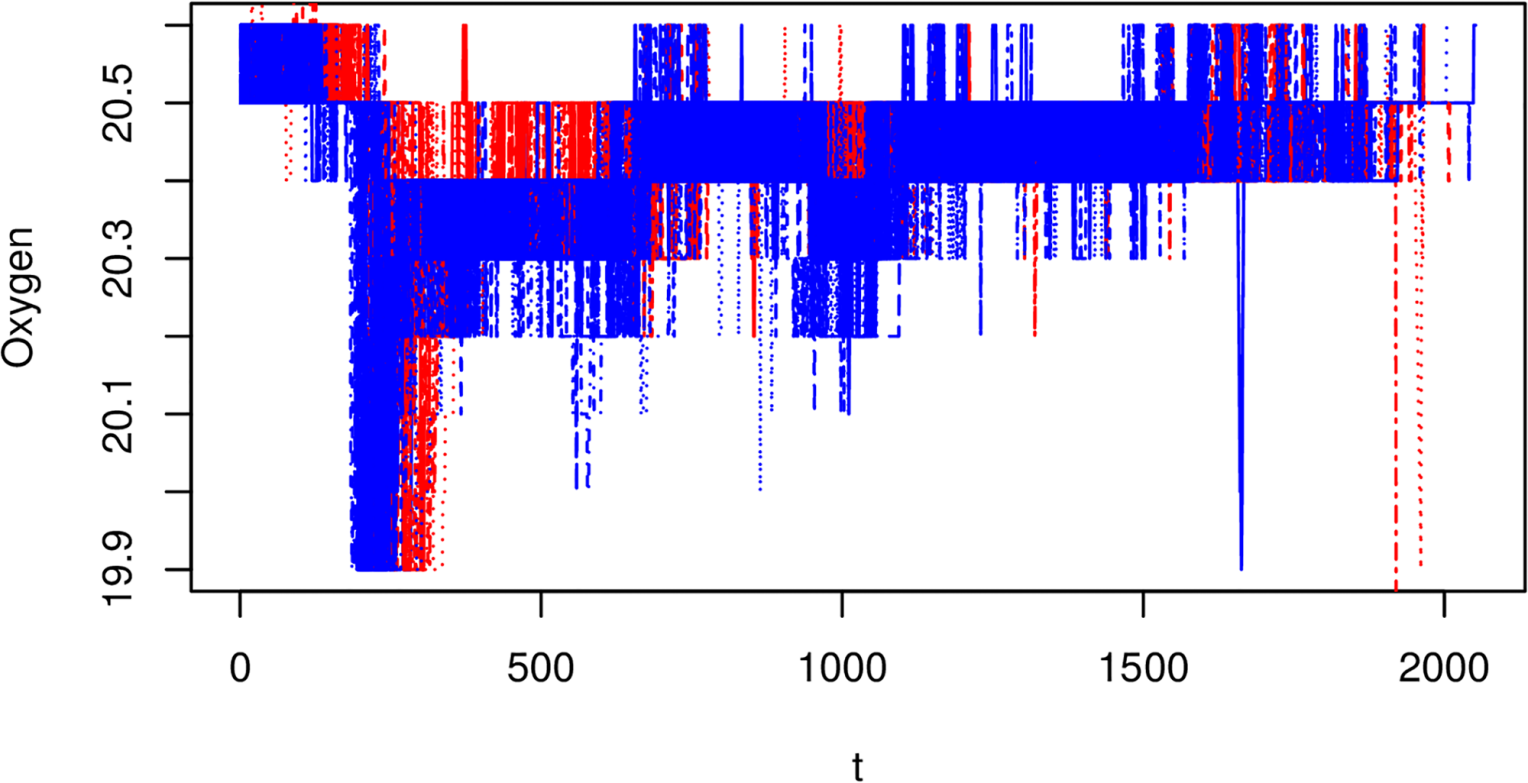
Raw oxygen data. Blue lines come from growing processes in group *G*_1_ and red lines from group *G*_2_. Note the x-axis reflects the time index for each 30min observation interval.

**Fig 6:**
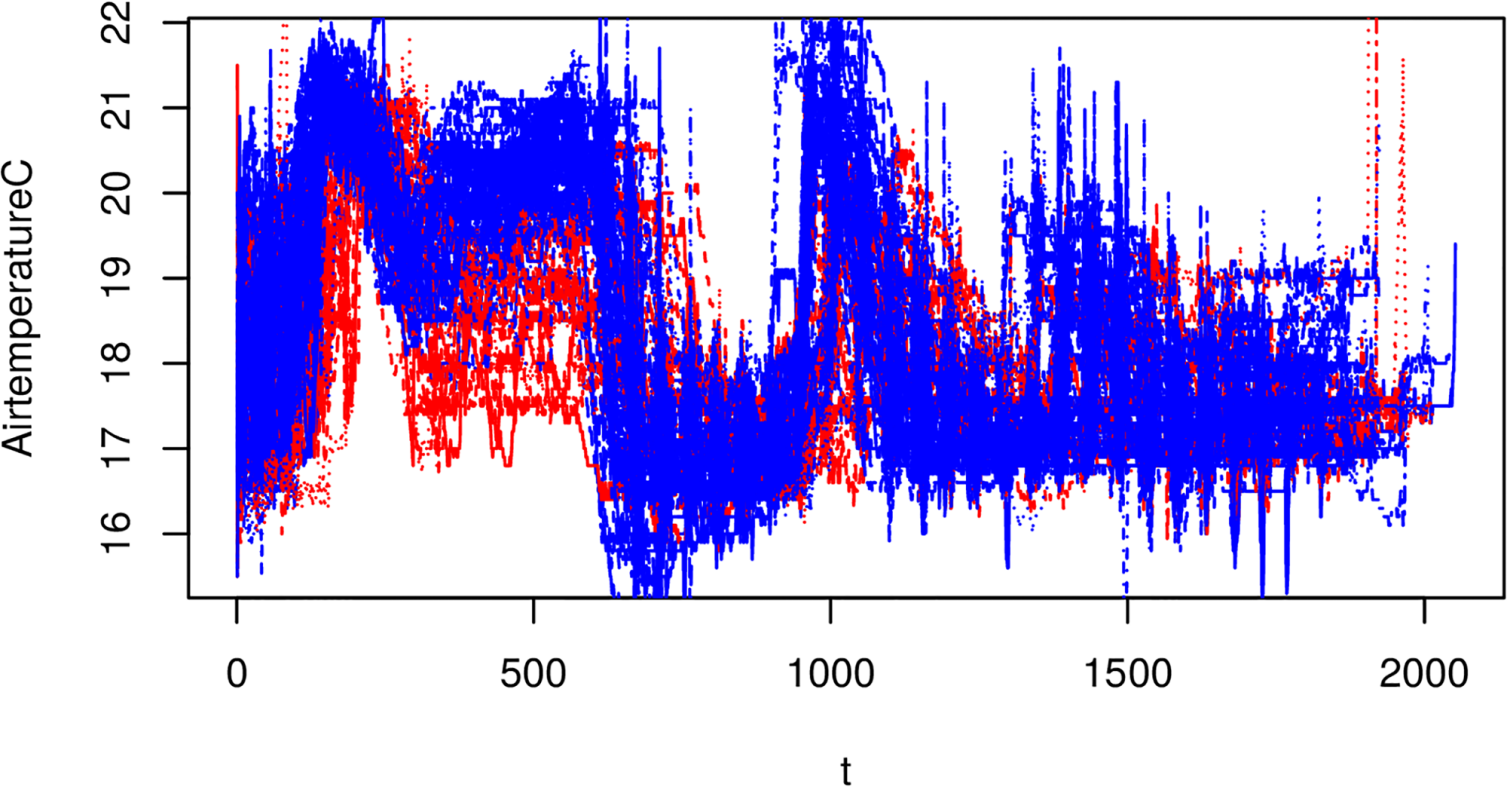
Raw temperature data. Blue lines come from growing processes in group *G*_1_ and red lines from group *G*_2_. Note the x-axis reflects the time index for each 30min observation interval.

We now start this section with a presentation of the raw functional covariate data, which we will be using as regressors in the VDFR. Figs 5 and 6 show for 92 growing processes, the evolution of the growing room air temperature and oxygen level from aeration to the last day of mushroom harvest. These schedules are fairly consistent across different growing processes and for the different types of compost, so we will treat this as a homogeneous sample.

**Fig 7:**
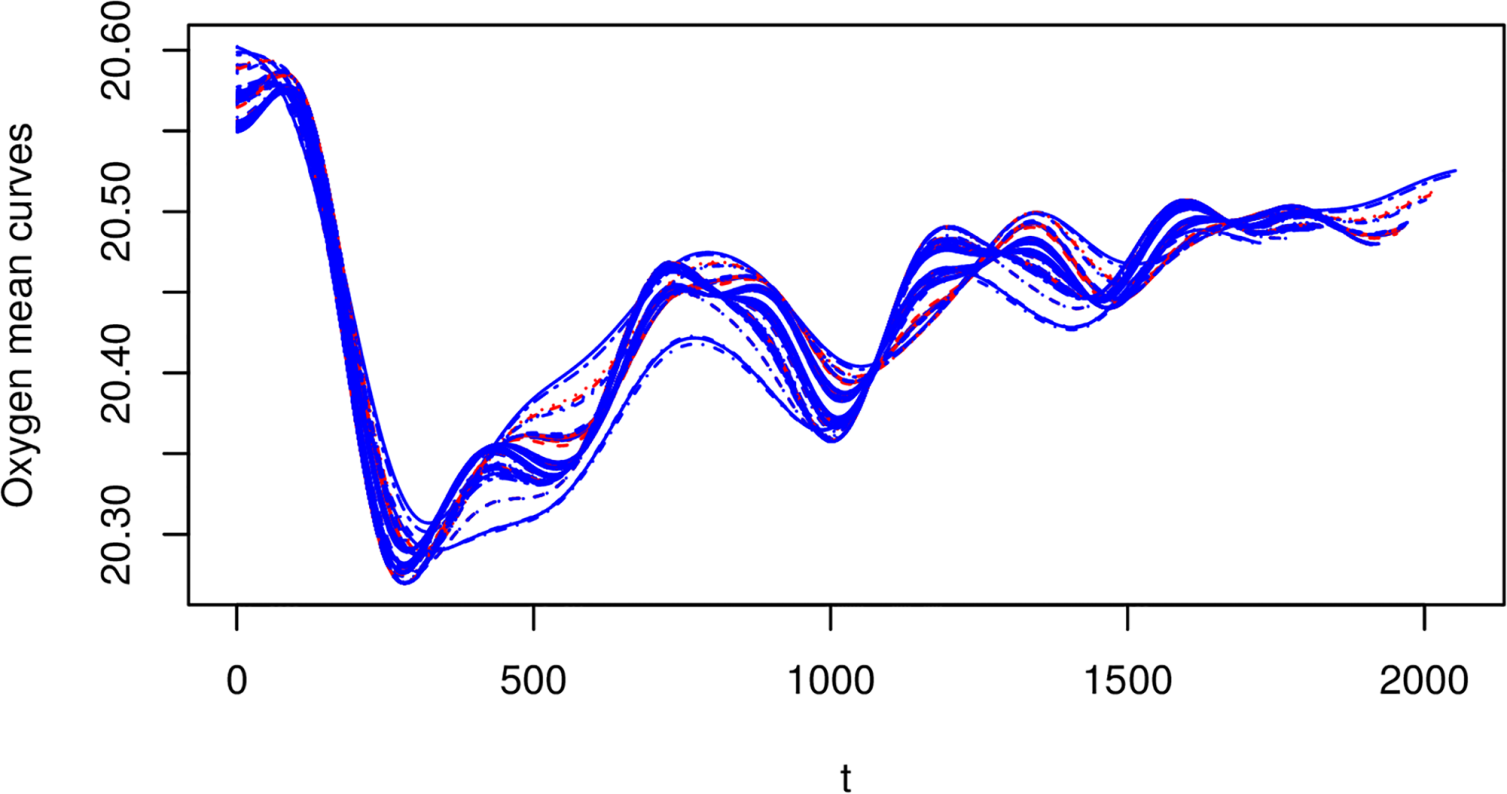
Mean oxygen data. Spline was fit with *K* = 15 knot points. Note the x-axis reflects the time index for each 30min observation interval.

**Fig 8:**
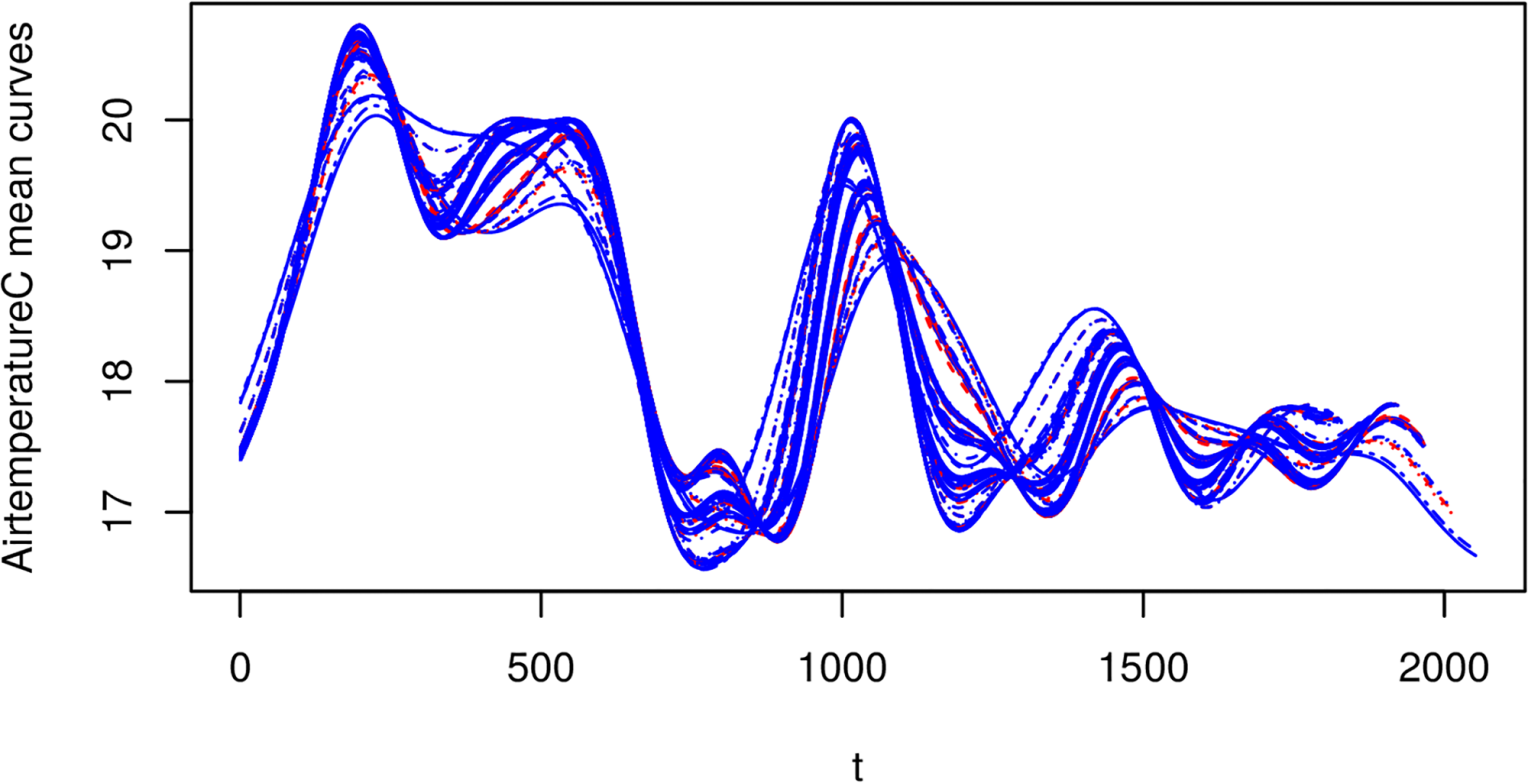
Mean temperature data. Spline was fit with *K* = 20 knot points. Note the x-axis reflects the time index for each 30min observation interval.

As in standard functional regression, we can demean the covariate function, but we now face an issue in that the (bivariate) mean function is not clearly defined. However, the conditional mean of *X*_*i*_(*t*) given *T*_*i*_, denoted *μ*_*X*_*i*_|*T*_(*t*), can be estimated using a GAM as follows:

$$\begin{array}{cc} X_{i}\left(t\right) & =\mu_{X_{i}|T}\left(t\right)+\epsilon_{t}\left(t,T_{i}\right)\\ g(\mu_{X_{i}|T}\left(t\right)) & =f\left(t,T_{i}\right)\;(\text{identity link}) \end{array}$$

where *ϵ*_*t*_(*t*, *T*_*i*_) ∼ *N*(0, *σ*^2^*I*) and *f* is a smooth function. The mean function will fall on the same trapezoidal domain {*t*, *T*_*i*_: min_*i*_ *T*_*i*_ ≤ *t* ≤ *T*_*i*_ ≤ max_*i*_ *T*_*i*_} described above. We recall that for a thin-plate regression spline, we do not need to select the location of the knot points, but we do need to select their number (*K*). Figs 7 and 8 show, the fitted mean curves for the oxygen and air temperature covariates.

### 5.1 Single functional covariate VDFR

**Fig 9:**
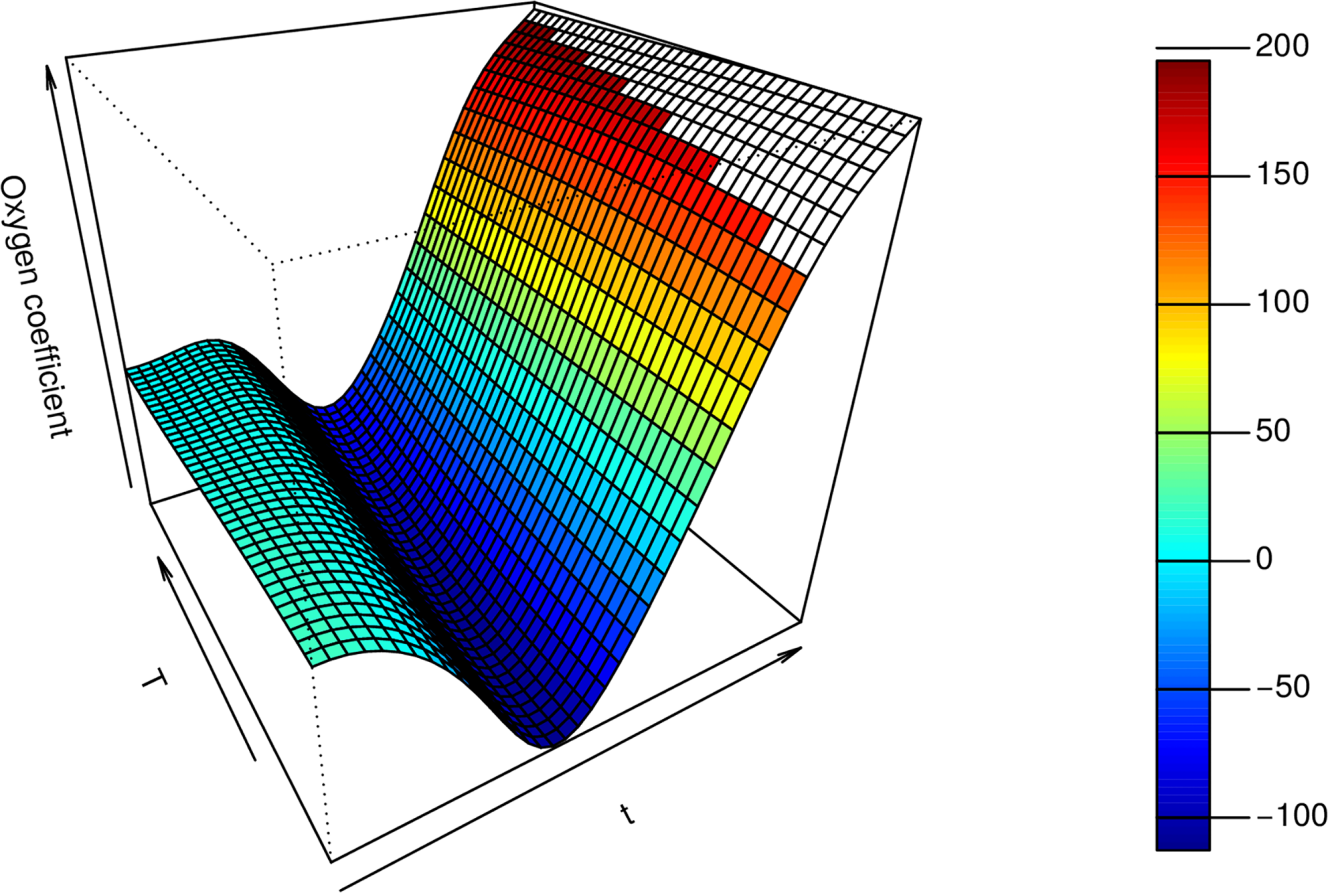
The estimated coefficient surface from the VDFR with a single functional regressor (oxygen). Note the t-axis reflects the time index for each 30min observation interval. The T-axis reflects the index of the number of days for which the production process was performed before harvesting for the given experiment.

The next step is to then fit the model (Eq 13) using the demeaned covariates, ie. the covariates haveing removed the average level. For clarity of explanation, we first report the results for VDFR with a single functional regressor and a single scalar covariate (Compostfilledkgsqm). Fig 9 presents the estimated coefficient surface across the *t*, *T* dimensions for the VDFR with the oxygen functional covariate. The first thing to note is that the estimated coefficient function is almost constant across the *T* dimension as we move to different values of *t*. This means that if we have growing processes of varying length *T*_*i*_, *i* ∈ 1 … *n*, the effect of the oxygen covariate at a point *t* < min(*T*_*i*_) will be similar across all growing processes.

**Fig 10:**
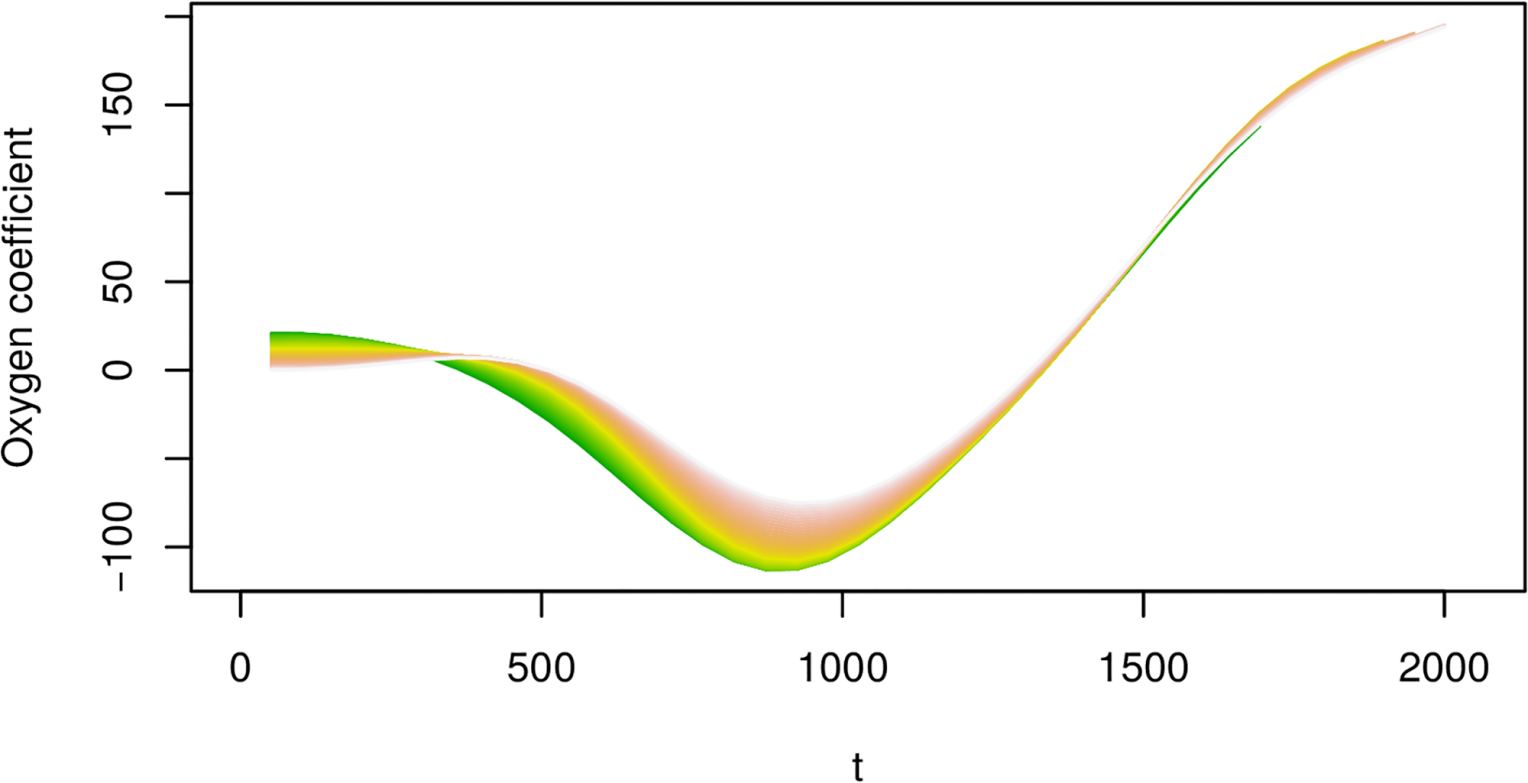
Cuts through the surface in Fig 9. Note the x-axis reflects the time index for each 30min observation interval.

For a concrete example, we look at Fig 10, which is formed by taking cuts in the *T* direction through the surface in Fig 9. We note the different coefficient curves, each of which is now a function of *t* only. The shortest line corresponds to a cut through a surface for the lowest value of *T* ≈ 1720. The longest line corresponds to a cut through a surface for the highest value of *T* ≈ 2050. If we look at the value of the coefficient function at *t* = 800, for example, this is very similar across the different curves, which means that the effect of the oxygen covariate at this point in the growing process is similar for both the shortest and longest growing processes.

Interpreting these curves now in terms of their effects on the yield, the portion of the coefficient curve which is negative indicates that an increase in the level of the covariate during these times is associated with a decrease in the average yield. Where the coefficient curve is positive, an increase in the level of the covariate during these times is associated with an increase in the average yield. Concretely, in the case of the oxygen covariate, the coefficient surface is below zero for values of *t* < 1200, and above zero for *t* > 1200. Under the model then, a modification of the oxygen schedule so that the oxygen level from the beginning of the growing process until *t* ≈ 1200 is decreased, followed by an increase after this point, will be associated with an increase in the average yield—for all values *T* of the growing process duration.

We present the results for the adjusted R^2^, deviance and REsidual Maximun Likelihood (REML) in Section 5.1.1. These measures of model performance are standard in regression contexts to assess the quality of the fit obtained, for a given model, see discussion in [11]. The analysis can be interpreted to demonstrate that Compostfilledkgsqm is generally found to be significant in the regression, as are the Oxygen and HumDeficitinletgkg functional covariates. The regression with the Oxygen functional covariate has a high adjusted R^2^ of over 20%, and we have seen from the explanation above that its effect on yield is interpretable throughout the growing process.

We should note that in choosing the number of knot points *K*, we aimed to maximize the explanatory power of the model, with a dual objective of keeping the model parsimonious. Our approach was to thus fit every VDFR single-covariate model (without considering the scalar covariate) multiple times (with *K* ∈ {5, 10, …, 50} and aimed to maximise the adjusted R^2^ metric (which penalises less parsimonious models) to determine the appropriate choice for *K*. These results, along with the respective deviance and REML values are presented in detail in the section 5.1.1.

#### 5.1.1 Results for knot number choice

**Fig 11:**
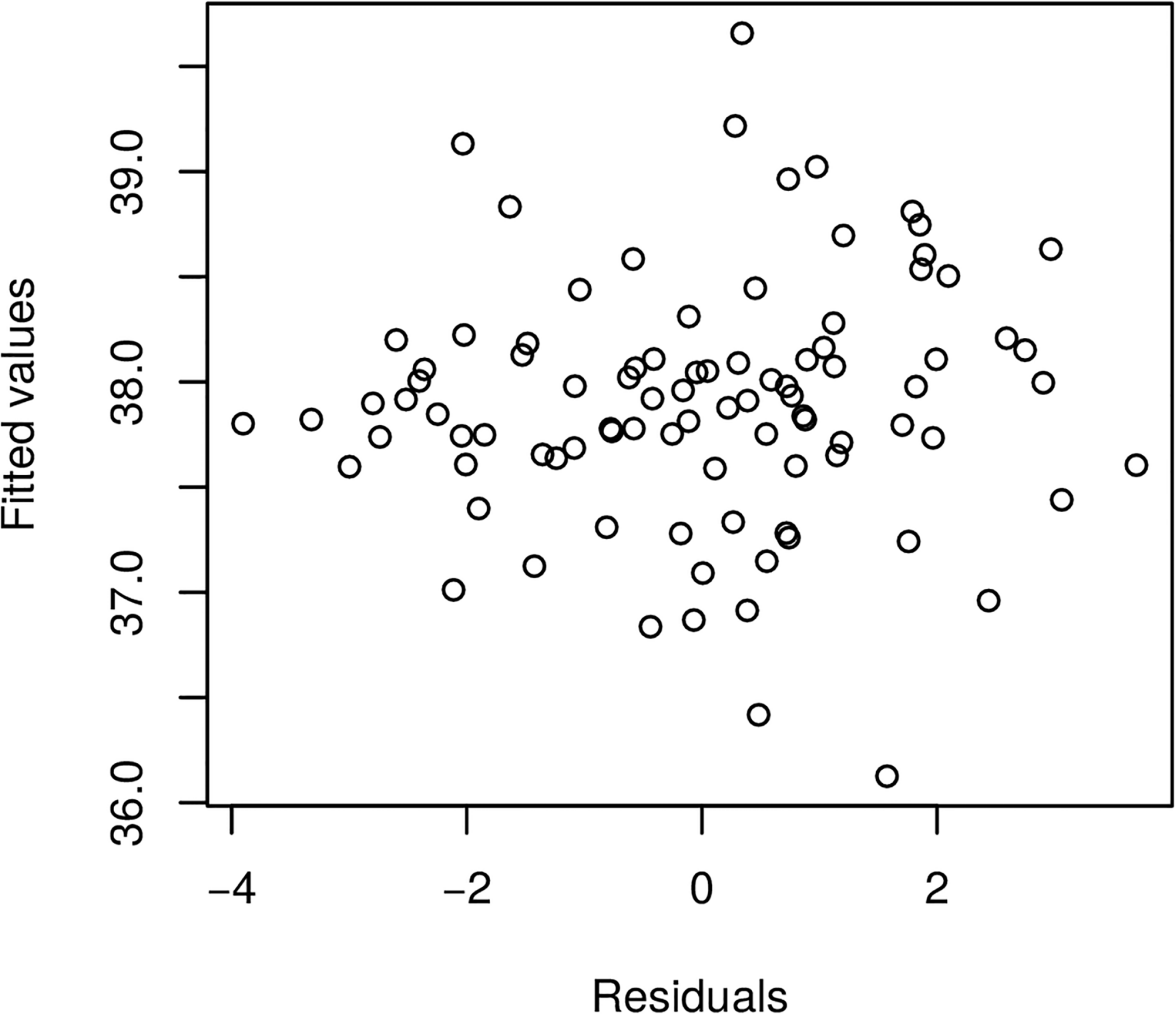
Raw residuals—Co2.

**Fig 12:**
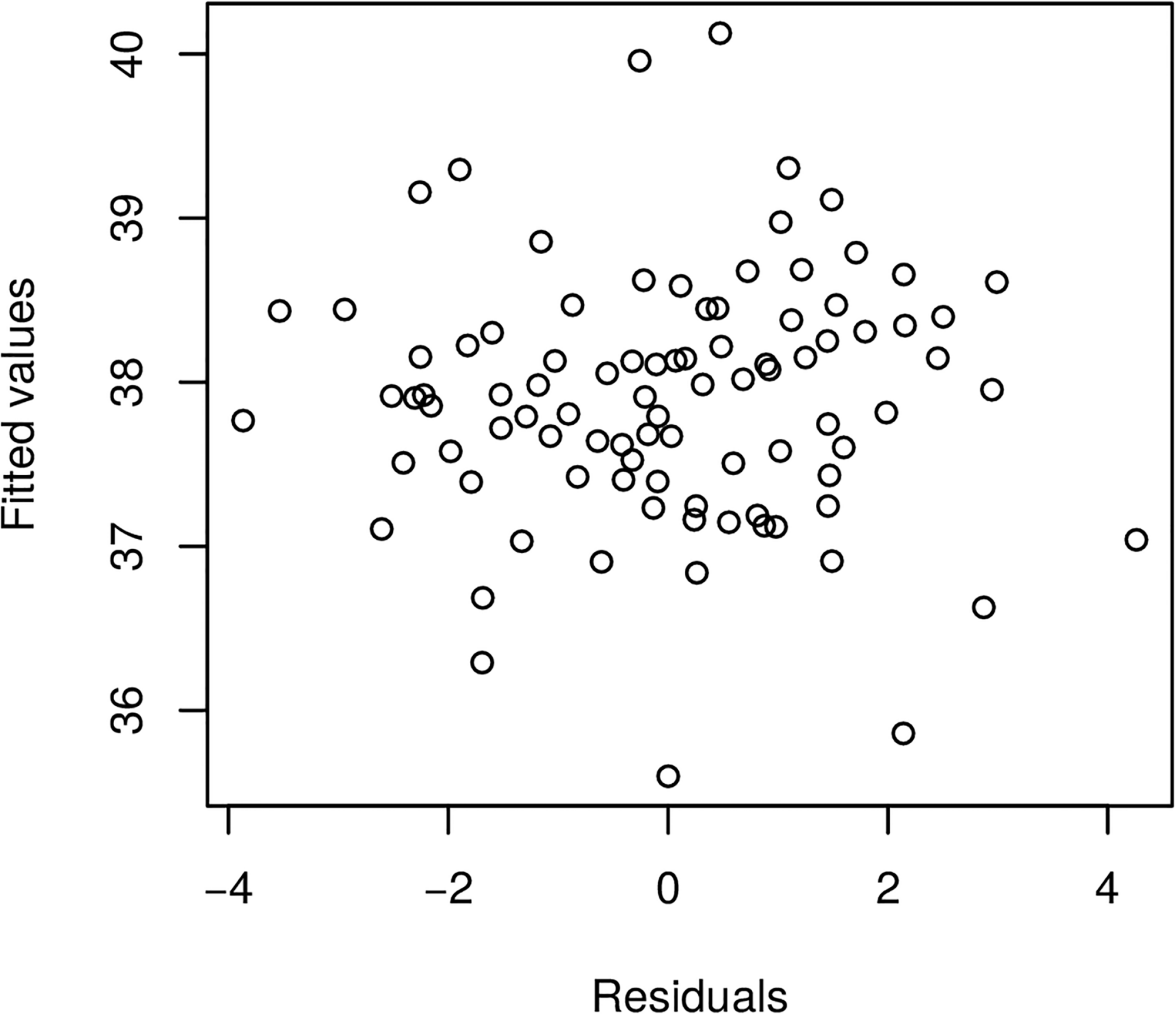
Raw residuals—Humidity deficit.

**Fig 13:**
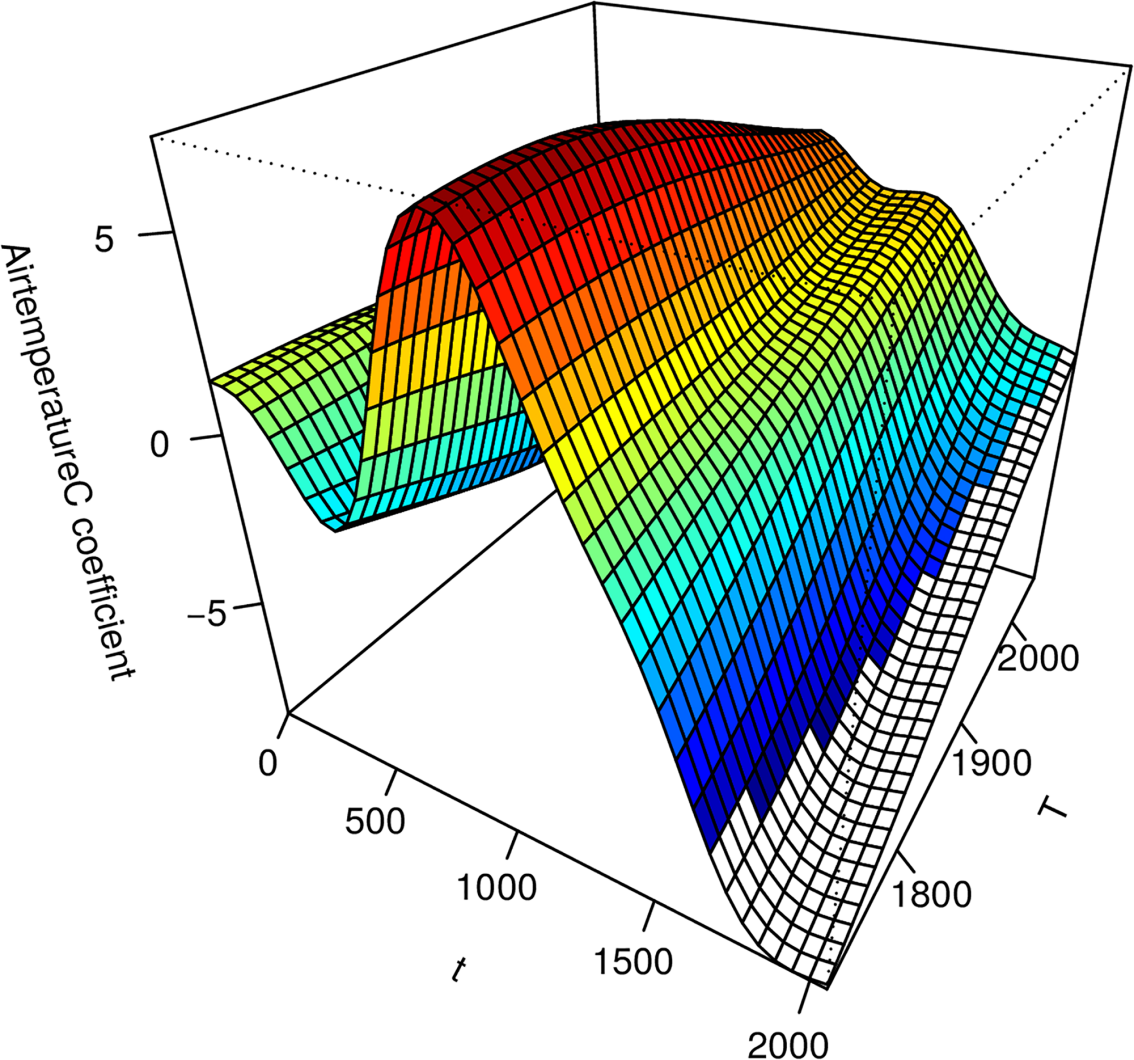
The estimated coefficient surface from the VDFR with a single functional regressor (air temperature). Note the t-axis reflects the time index for each 30min observation interval. The T-axis reflects the length of time before harvest for the given growing trial.

**Fig 14:**
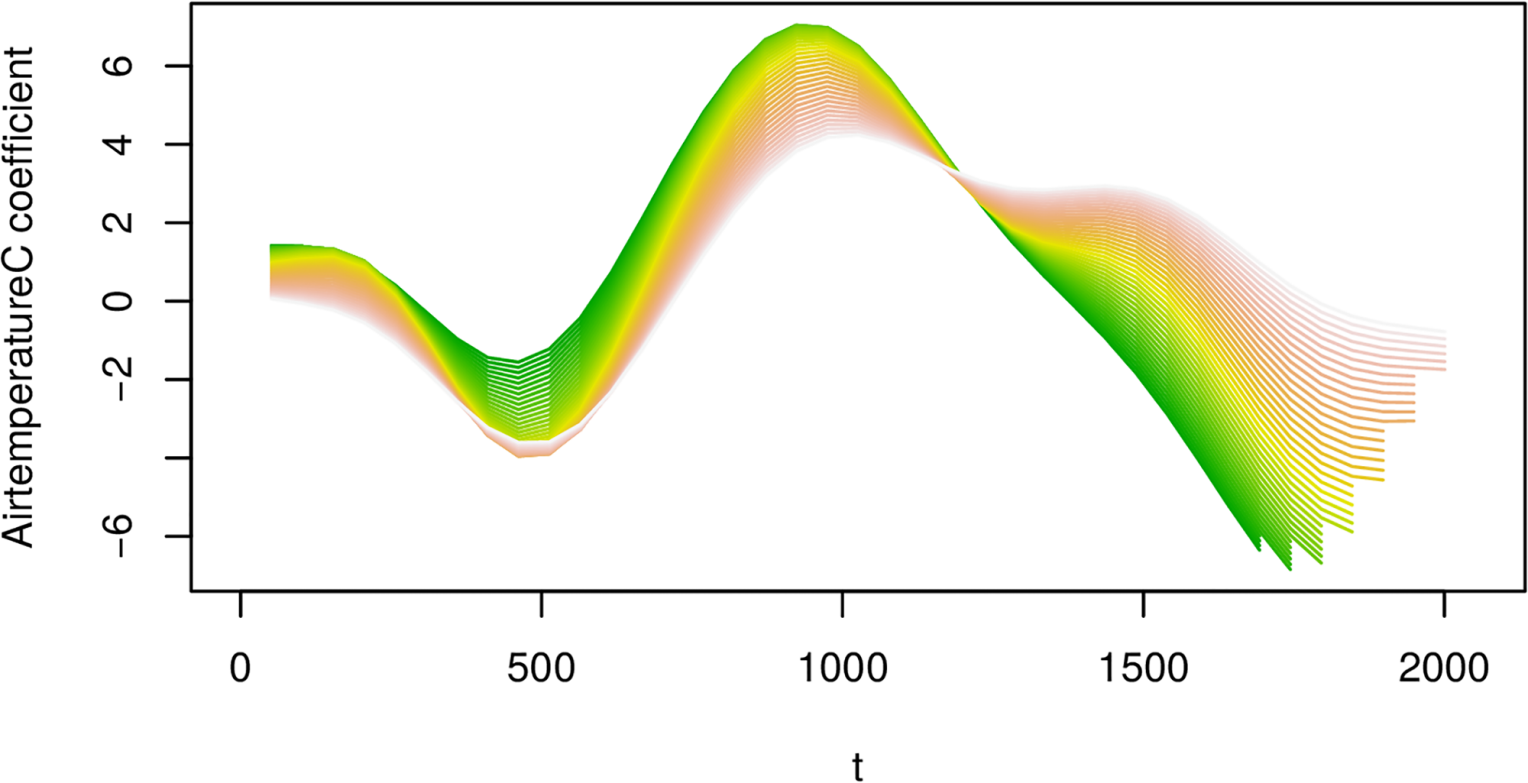
Cuts through this surface of the estimated coefficient surface from the VDFR with a single functional regressor (air temperature). Note the t-axis reflects the time index for each 30min observation interval. The T-axis reflects the length of time before harvest for the given growing trial.

**Fig 15:**
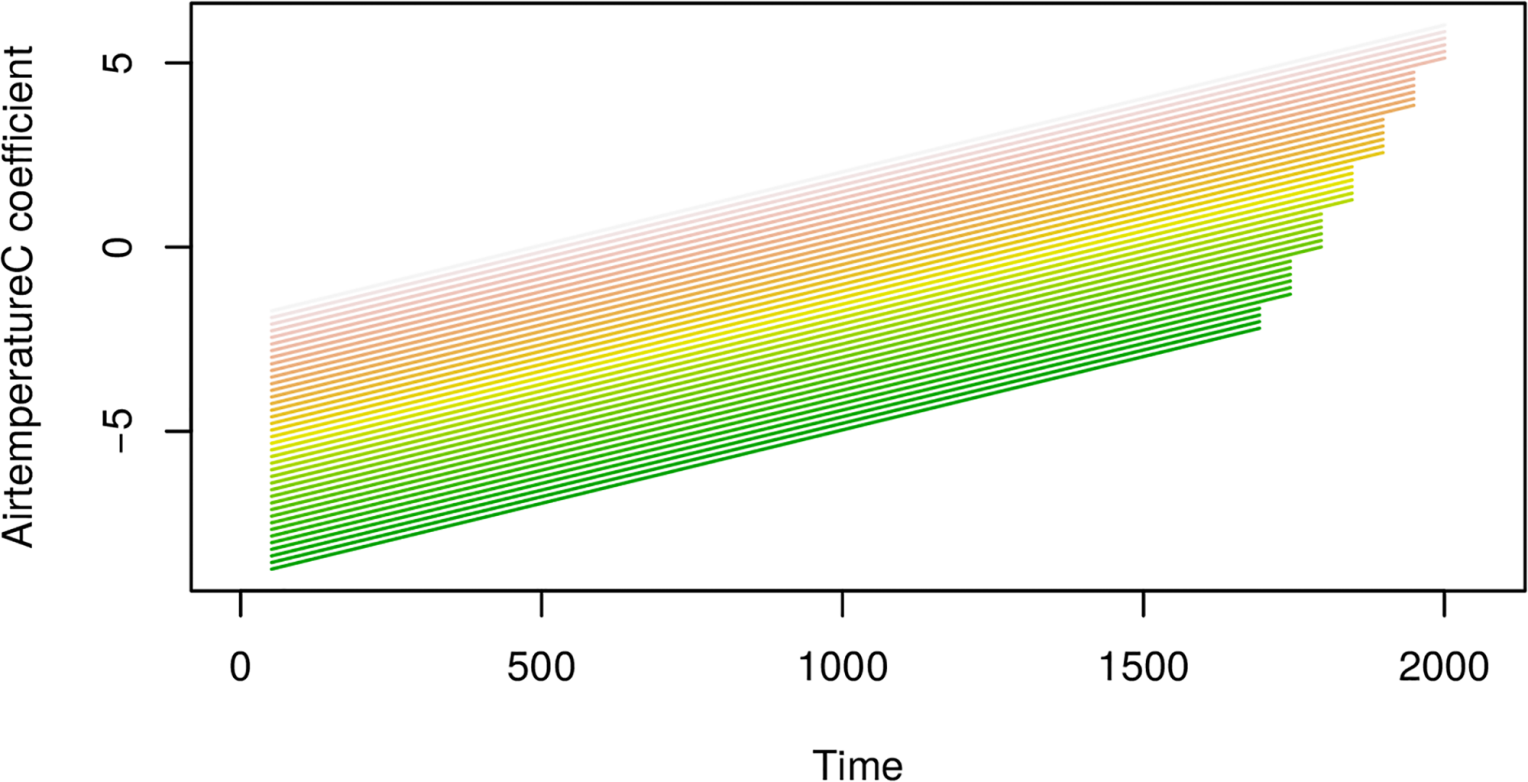
Cuts through the estimated coefficient surface for airtemperature from the VDFR with two functional regressors (airtemperature and oxygen). Adjusted r-squared for this regression is 0.175. Note the x-axis reflects the time index for each 30min observation interval.

**Fig 16:**
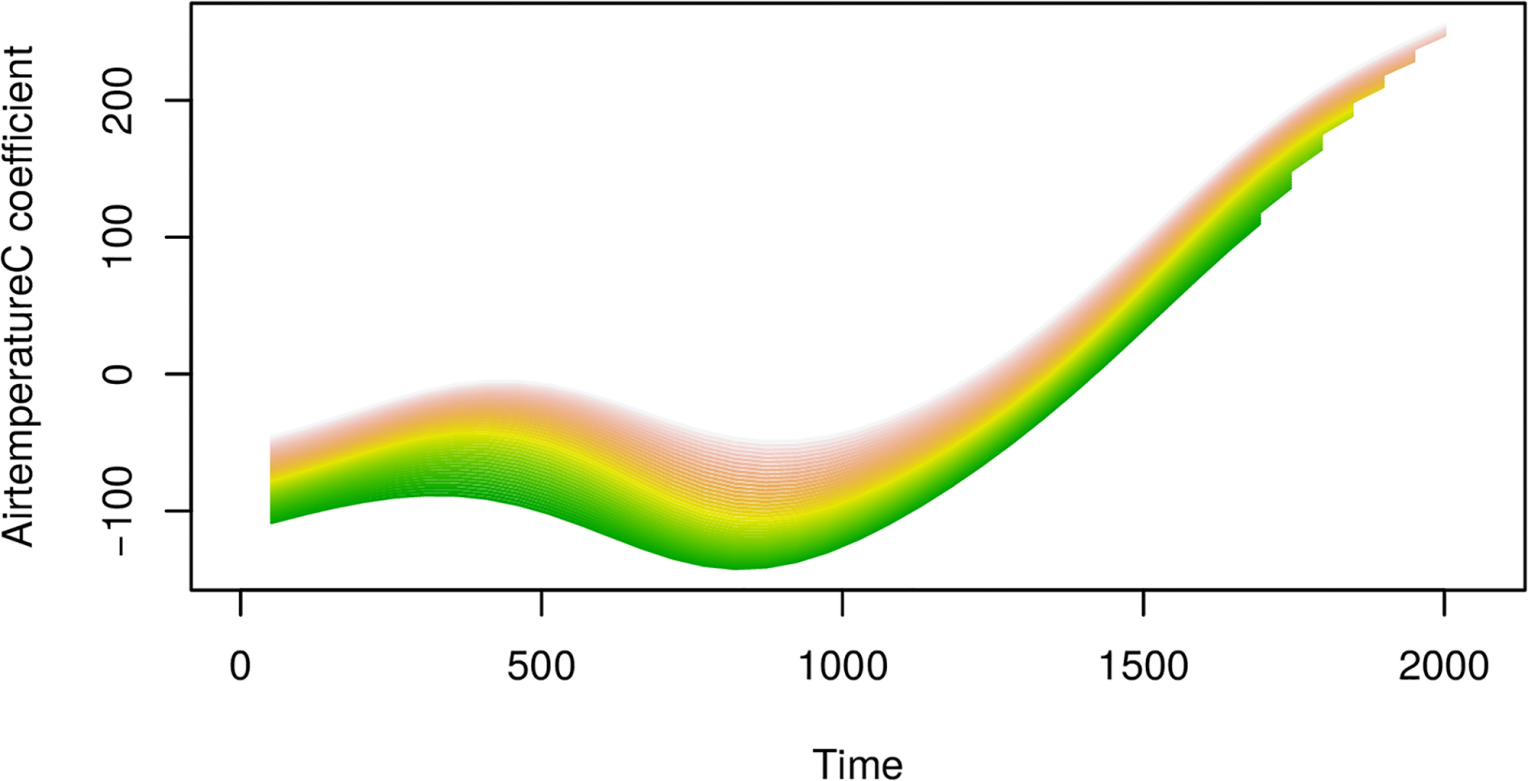
Cuts through the estimated coefficient surface for oxygen from the VDFR with two functional regressors (airtemperature and oxygen). Adjusted r-squared for this regression is 0.175. Note the x-axis reflects the time index for each 30min observation interval.

We fit the VDFR model and estimate both the coefficient function and smoothing parameters via restricted likelihood (REML), see details in [44]. The results of this analysis are presented in Tables 4, 5, 6, 7 and 8. Furthermore, they are displayed visually in Figs 11, 12, 13,14, 15 and 16.

**Table 4:** Results for VDFR with a single functional regressor (air temperature) for different choices of *K*.

K	R-squared (adj)	Deviance explained	REML
5	0.017	0.062	179.0
10	0.065	0.123	178.2
15	0.107	0.178	177.9
20	0.101	0.173	178.0
25	0.095	0.166	178.0
30	0.098	0.170	178.0
35	0.101	0.174	178.0
40	0.099	0.172	178.0
45	0.099	0.172	178.0
50	0.099	0.172	178.0

**Table 5:** Results for VDFR with a single functional regressor (evaporation) for different choices of *K*.

	R-squared (adj)	Deviance explained	REML
5	-0.030	0.004	190.3
10	-0.030	0.004	190.3
15	-0.030	0.004	190.3
20	-0.030	0.004	190.3
25	-0.030	0.004	190.3
30	-0.030	0.004	190.3
35	-0.030	0.004	190.3
40	-0.030	0.004	190.3
45	-0.030	0.004	190.3
50	-0.030	0.004	190.3

**Table 6:** Results for VDFR with a single functional regressor (co2 production) for different choices of *K*.

	R-squared (adj)	Deviance explained	REML
5	0.086	0.128	182.9
10	0.084	0.129	183.2
15	0.085	0.131	183.2
20	0.090	0.139	183.1
25	0.091	0.141	183.1
30	0.091	0.141	183.1
35	0.091	0.141	183.1
40	0.091	0.141	183.1
45	0.091	0.142	183.1
50	0.091	0.142	183.1

**Table 7:** Results for VDFR with a single functional regressor (oxygen) for different choices of *K*.

	R-squared (adj)	Deviance explained	REML
5	0.114	0.156	167.3
10	0.126	0.176	167.1
15	0.138	0.192	166.9
20	0.137	0.192	167.0
25	0.136	0.192	167.0
30	0.136	0.192	167.0
35	0.137	0.193	167.0
40	0.136	0.193	167.0
45	0.136	0.192	167.0
50	0.136	0.192	167.0

**Table 8:** Results for VDFR with a single functional regressor (HumDeficitinletgkg) for different choices of *K*.

	R-squared (adj)	Deviance explained	REML
5	0.126	0.155	175.7
10	0.126	0.155	175.7
15	0.126	0.155	175.7
20	0.126	0.155	175.7
25	0.126	0.155	175.7
30	0.126	0.155	175.7
35	0.126	0.155	175.7
40	0.126	0.155	175.7
45	0.126	0.155	175.7
50	0.126	0.155	175.7

Table 9 provides the results for the fitting performance results for the VDFR models with a single functional covariate and a single scalar covariate (Compostfilledkgsqm). Then in Table 10 summarises the optimal choices for *K* for each functional covariate. We do not consider the evaporation covariate further as it was not found to have explanatory power for the yield.

**Table 9:** The results for the VDFR models with a single functional covariate (in the table) and a single scalar covariate (Compostfilledkgsqm), for which the estimated coefficient value is provided in the table. Asterisks indicate that the covariate was found to be significant at the 5% level in the model, while the symbol ^†^ that it was significant at the 10% level. Deviance column stands for the explained deviance.

	R-squared (adj)	Deviance	REML	Compostfilledkgsqm
AirtemperatureC	0.148	0.223	177.1	0.14*
Co2productionghm2	0.090	0.148	184.5	0.06
Oxygen*	0.213	0.272	164.4	0.17*
HumDeficitinletgkg*	0.148	0.186	176.0	0.10^†^

**Table 10:** Optimal choices for the number of knots *K* for the VDFR with a single functional regressor.

Covariate	K
Air temperature	15
Oxygen	15
Humidity deficit	5
CO_2_ production	25

**Fig 17:**
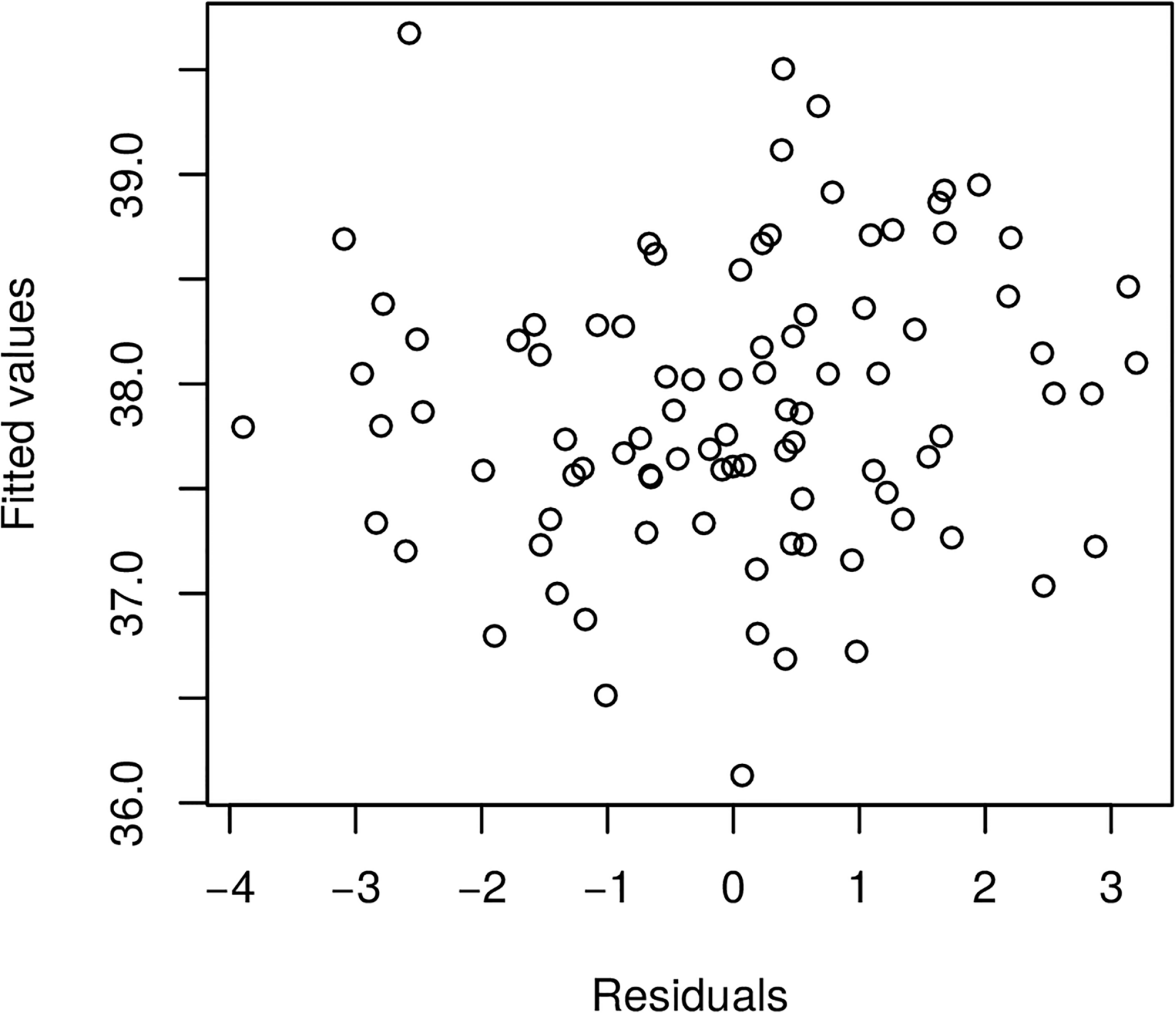
Raw residuals—Airtemperature.

**Fig 18:**
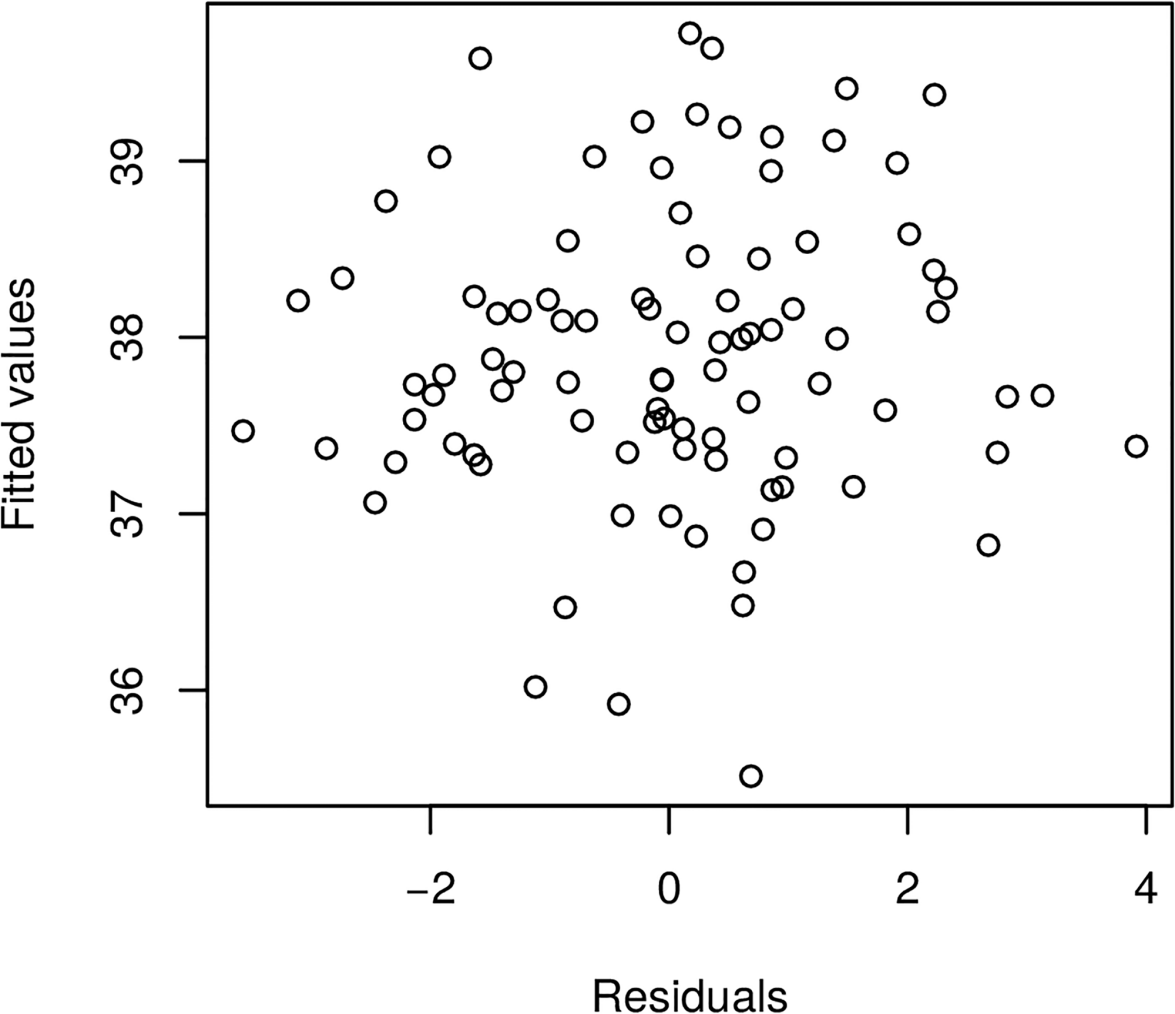
Raw residuals—Oxygen.

Furthermore, we present in Figs 17 and 18 the model fitted residuals for two key covariate functional inputs corresponding to air temperature and Oxygen content. We note that these residual plots demonstrate that the model assumptions are suitably satisfied, since the residuals have sample properties that one would expect from a well performing regression model. For instance, there is no residual evident trend structure and there is no evident serial correlations.

### 5.2 Multiple covariate VDFR and model selection

We can generalise Eq 13 so that we can consider multiple functional covariates as follows:

$$\begin{array}{c} g\left(\mu_{i}\right)=\beta_{0}+Z\alpha_{i}+\Sigma_{j=1}^{p}\frac{1}{T_{i}}\int_{0}^{T_{i}}X_{i,j}\left(t\right)\beta_{j}\left(t,T_{i}\right)dt \end{array}$$ (18)

We consider the model with *j* = 2, 3, 4, i.e. with combinations of the 4 functional covariates (air temperature, oxygen, humidity deficit and CO_2_ production), omitting the evaporation covariate which was not found to be informative. Since this application only requires a small number of covariates, we perform an exhaustive search across the model space, i.e. the total number of models we consider is $(\begin{array}{c} 4\\ 2 \end{array})+(\begin{array}{c} 4\\ 3 \end{array})+(\begin{array}{c} 4\\ 4 \end{array})=11$ models. We note that in the following, we will use the optimal choices for *K* identified in the univariate VDFR in the previous section.

We present the results for the bivariate VDFR model in Tables 10 and 11, for the trivariate model in Table 12. We also fit the full (4-covariate) model, for which the adjusted R^2^ value was 0.276, but none of the functional or scalar covariates found to be significant. The highest explanatory power was found to be in the model with CO_2_ production, Oxygen and HumDeficitinletgkg as the functional covariates. Interestingly, when CO_2_ production and Oxygen are both part of the regression model, only one of the two is found to be significant. This has a physical interpretation, as Oxygen displaces CO_2_ in the growing room.

**Table 11:** The results for the bivariate VDFR models. Asterisks indicate that the covariate was found to be significant at the 5% level, whereas the daggers indicate significance at the 10% level.

Covariate 1	Covariate 2	Adjusted R^2^	Compostfilledkgsqm
AirtemperatureC*	Co2productionghm2	0.295	0.107^†^
AirtemperatureC	Oxygen*	0.214	0.146*
AirtemperatureC	HumDeficitinletgkg*	0.249	0.121*
Co2productionghm2	Oxygen*	0.241	0.127*
Co2productionghm2^†^	HumDeficitinletgkg*	0.237	0.06
Oxygen*	HumDeficitinletgkg*	0.287	0.122*

**Table 12:** The results for the trivariate VDFR models. Note, CF is corresponding to Compostfilledkgsqm which is in units of kg per sqm. Asterisks indicate that the covariate was found to be significant at the 5% level in the model, while the symbol ^†^ that it was significant at the 10% level.

Cov. 1	Cov. 2	Cov. 3	Adjusted R^2^	CF
AirtemperatureC	Co2productionghm2	Oxygen*	0.26	0.15*
AirtemperatureC	Co2productionghm2	HumDeficitinletgkg*	0.23	0.10^†^
AirtemperatureC	Oxygen*	HumDeficitinletgkg*	0.30	0.14*
Co2productionghm2	Oxygen*	HumDeficitinletgkg*	0.31	0.13*

## 6 Cost optimisation analysis for controling growing environment to maximize expected yield

In this section we consider how to adjust a farmer’s particular environmental condition schedule, in order to maximize the yield obtained when harvesting the mushroom crop. The optimal schedule will be obtained based on the model developed from the variable domain functional regression. We will focus only on optimizing the production considering the functional covariates. This is reasonable, since for a commercial grower, the scalar covariates would typically be compost-related and may include those listed in Table 2. The grower would have no control over these covariates, unless they produced their own compost. Of course, the results presented would be of research interest to companies specialised in compost preparation also.

In presenting this framework we assume that the grower has a precision agriculture environment with sensors monitoring the growing process and the ability to alter the growing environment variables such as: air temperature, oxygen and CO_2_ levels, and the evaporation conditions for moisture through the humidity deficit. For this purpose, however, we also need to rely on commercial grower experience in setting out constraints for the maximum deviations from existing schedules for the growing environment. If we consider the example of the air temperature, increasing or decreasing temperatures at particular points in the growing process may have adverse effects on mushroom quality, and the mushroom may even die in excessively high temperatures, therefore limits on the available range of covariates should be applied.

We present the results in the context where we assume the growing period is known. Note, if the actual growing period is unknown *a priori* the procedure to be specified can be trivially adapted. We assume that the grower has *p* variables that are important in determining the growing conditions and therefore also the ultimate yield of product obtained in a growing period [0, *T*]. Furthermore, we assume that based on previous historical realizations of growing environment, that one has obtained a calibration of the variable domain functional regression model which is estimated using these historical records in order to obtain the best fitting model with *p* functional covariates. This provides a model for the expected yield at time *T* given by the fitted VDFR as follows:

$$\begin{array}{c} \hat{y}={\hat{\alpha }}_{0}+\sum_{k=1}^{q}{\hat{\alpha }}_{k}z_{i,k}+\sum_{j=1}^{p}\frac{1}{T}\int_{0}^{T}{\hat{\beta }}_{j}\left(t,T\right)x_{i,j}\left(t\right)dt. \end{array}$$ (19)

We also assume that a grower has a particular current preference for the schedule at which the covariates (environmental control variables {*x*_*T*, *j*_(*t*)}_*j*=1:*p*,*t*∈[0,*T*]_) are typically set for a growing period of length [0, *T*], however these are not designed to optimize the expected yield and instead typically based on practical experience.

In this section we will therefore define a new schedule for each environmental factor that will be obtained to maximize the expected yield for growing period [0, *T*]. We define the new schedule of these environmental factors according to process ${\left\{{\tilde{x}}_{T,j}\left(t\right)\right\}}_{j=1:p,t\in \left[0,T\right]}$ and we assume that the new schedule can be represented for each environmental control factor according to piecewise constant levels. We believe this is practically meaningful as typically it will not produce good environmental growing conditions if growing conditions are constantly changing in time, instead in practice it is better to have stable conditions for fixed periods of time, which in general may be irregularly spaced, and delineated by adjustment times 0 < *t*_1_ < *t*_2_ < … < *t*_*n*_ < *T*. This produces the new schedule of the environmental factor, that we parametrize, for the *j*-th covariate, by the piecewise representation

$$\begin{array}{c} {\tilde{x}}_{T,j}\left(t\right)=\sum_{i=1}^{n}\gamma_{i,j}I_{t\in [t_{i},t_{i+1})}\left(t\right)\; \end{array}$$ (20)

for $\gamma_{1,j},\ldots ,\gamma_{n,j}\in R^{n}$. For convenience, we will assume that all environmental variables are altered on the same time schedules, it is trivial to relax this assumption.

Furthermore, we define a piece-wise cost function that measures the costs associate with deviation, in intervals {[*t*_*i*_, *t*_*i*+1_)}_*i*=1:*n*_, from the current farming environmental schedule. We denote the cost for altering the current schedule in time [*t*_*i*_, *t*_*i*+1_) for the *j*-th covariate from *x*_*T*,*j*_(*t*) to the new value *γ*_*i*,*j*_ as quantified by a function $C_{i,j}({\tilde{x}}_{T,j}\left(t\right),x_{T,j}\left(t\right))$, where *x*_*T*,*j*_(*t*) is the current practice of the farmer. In practice, this cost function is some mapping for instance of the additional consumed energy, labor, wear and tear of device etc. required to change the physical environment to the new environmental condition, such quantities can be quantified in dollars in practice. Then the total cost of altering all *p* covariates in the *n* schedule intervals in which adjustments may be considered to *p* different environmental controls is given by:

$$\begin{array}{c} C_{T}^{\text{Total}}\left(p\right)=\sum_{i=1}^{n}\sum_{j=1}^{p}C_{i,j}({\tilde{x}}_{T,j}\left(t\right),x_{T,j}\left(t\right)). \end{array}$$ (21)

Furthermore, we will assume that the grower has specifications based on experience that they would never allow the growing environment conditions to enter, these are grower specific constraints which will depend on the specific country’s climate. Climate affects energy consumption, production and the growing environment, and the resulting constraints manifest as maximal ranges of deviation for ${\tilde{x}}_{T,j}\left(t\right)$ relative to *x*_*T*,*j*_(*t*), given by constraint piece-wise envelopes ${\tilde{x}}_{T,j}\left(t\right)\in [x_{T,j}\left(t\right)-x_{m}in\left(t\right),x_{T,j}\left(t\right)+x_{m}ax\left(t\right)]$, for each time segment [*t*_*i*_, *t*_*i*+1_). To encode these specifications we assume that the cost of exceeding these user specified constraints on the environmental conditions is infinite i.e. for any *i*, *j* one has that $C_{i,j}({\tilde{x}}_{T,j}\left(t\right),x_{T,j}\left(t\right))=\infty$ if ${\tilde{x}}_{T,j}\left(t\right)\;\notin \;[x_{T,j}\left(t\right)-x_{min}\left(t\right),x_{T,j}\left(t\right)+x_{max}\left(t\right)]$

Now the goal of this section is to consider the optimal selection of sequences {*γ*_*i*,*j*_}_*i*=1:*n*,*j*=1:*p*_ subject to a given total budget specified by constraint $C_{T}^{\text{Total}}\left(p\right)\leq C_{\text{max}}$. This allows us to therefore form the following optimization problem to determine the optimal deviations of schedule from current growing practices, to maximize expected yield, based on the VDFR model.

Select the optimal environmental condition controls, denoted by ${\left\{\gamma_{i,j}^{\text{opt}}\right\}}_{i=1:n,j=1:p}$, subject to the solution given by

$$\begin{array}{ll} {\left\{\gamma_{i,j}^{\text{opt}}\right\}}_{i=1:n,j=1:p} & =\underset{{\left\{\gamma_{i,j}\right\}}_{i=1:n,j=1:p}}{\text{arg}\;\text{max}}{\hat{\alpha }}_{0}+\sum_{k=1}^{q}{\hat{\alpha }}_{k}z_{i,k}+\frac{1}{T}\sum_{j=1}^{p}\sum_{i=1}^{n}\gamma_{i,j}\int_{t_{i}}^{t_{i+1}}{\hat{\beta }}_{j}\left(t,T\right)dt.\\ & \;\text{s.t.}\;C_{T}^{\text{Total}}\left(p\right)=C_{\text{max}}. \end{array}$$ (22)

### 6.1 Example: Single covariate VFDR controlled environment to maximize expected yield

To illustrate an example of solving this objective function we will make the following model where we consider a single covariate VFDR structure such that *p* = 1 (allowing us to drop the *j* subindex in the example) and the removal of the non-functional covariates can be performed without loss of generality as explained previously, at least for the applications of interest to this manuscript.

We consider a cost of adjustment in each segment given by a quadratic function of the difference between the growers current schedule and the new target schedule at time *t*_*i*_, therefore taking the form

$$\begin{array}{c} C_{i}\left({\tilde{x}}_{T}\left(t\right),x_{T}\left(t\right)\right)={\left({\tilde{x}}_{T}\left(t\right)-x_{T}\left(t\right)\right)}^{2},\;\forall i\in \left\{1,\ldots ,n\right\}, \end{array}$$ (23)

when ${\tilde{x}}_{T}\left(t_{i}\right)\in [x_{T}\left(t_{i}\right)-x_{min}\left(t_{i}\right),x_{T}\left(t_{i}\right)+x_{max}\left(t_{i}\right)]$, such that the total cost is then given by

$$\begin{array}{c} C_{T}^{\text{Total}}=\sum_{i=1}^{n}C_{i}({\tilde{x}}_{T}\left(t\right),x_{T}\left(t\right)). \end{array}$$ (24)

Furthermore, we will consider a slightly modified version in which the total cost at each time point is constrained, so as to ensure a controlled effort over the whole growing period where we consider the modified constraint that $C_{i}({\tilde{x}}_{T}\left(t\right),x_{T}\left(t\right))={\tilde{C}}_{i}$ for each time *t*_*i*_ such that $\sum_{i=1}^{n}{\tilde{C}}_{i}=C_{\text{max}}$.

Solving this problem can be achieved under a Lagrange multiplier framework with a Lagrangian function given by

$$\begin{array}{ll} L\left(\lambda_{1},\ldots ,\lambda_{n},\gamma_{1},\ldots ,\gamma_{n}\right) & =\frac{1}{T}\sum_{i=1}^{n}\gamma_{i}\int_{t_{i}}^{t_{i+1}}\hat{\beta }\left(t,T\right)dt+\sum_{i=1}^{n}\lambda_{i}C_{i}\left({\tilde{x}}_{T}\left(t\right),x_{T}\left(t\right)\right)\\ & =\frac{1}{T}\sum_{i=1}^{n}\gamma_{i}\int_{t_{i}}^{t_{i+1}}\hat{\beta }\left(t,T\right)dt+\sum_{i=1}^{n}\lambda_{i}\left[{\left(\gamma_{i}-x_{T}\left(t_{i}\right)\right)}^{2}-C_{i}\right] \end{array}$$ (25)

where λ_1_, …, λ_*n*_ are the Lagrangian multipliers. We then solve the problem given by **∇_*γ*, λ_** = **0**.

In this case we find the system of equations given by

$$\begin{array}{ll} \frac{\partial L\left(\lambda ,\gamma \right)}{\partial \gamma_{i}} & =\frac{1}{T}\int_{t_{i}}^{t_{i+1}}\hat{\beta }\left(t,T\right)dt+2\lambda_{i}\left(\gamma_{i}-x_{T}\left(t_{i}\right)\right)=0,\;\forall i\in \left\{1,2,\ldots ,n\right\}\\ \frac{\partial L\left(\lambda ,\gamma \right)}{\partial \lambda_{i}} & ={\left(\gamma_{i}-x_{T}\left(t_{i}\right)\right)}^{2}-{\tilde{C}}_{i}=0,\;\forall i\in \left\{1,2,\ldots ,n\right\}. \end{array}$$ (26)

Via substitution it is then trivial to solve this system of equations to produce optimal solutions given by

$$\begin{array}{c} \begin{array}{cc} & \gamma_{i}=\pm \sqrt{{\tilde{C}}_{i}}+x_{T}\left(t_{i}\right).\\ & \lambda_{i}=-\frac{\int_{t_{i}}^{t_{i+1}}\hat{\beta }\left(t,T\right)dt}{2T(\pm \sqrt{{\tilde{C}}_{i}}+x_{T}\left(t_{i}\right)-x_{T}\left(t_{i}\right))} \end{array} \end{array}$$ (27)

One then just selects the combination of solutions that will maximize the objective function to obtain the required schedule in closed form. In future works we will seek additional optimality considerations such as shelf life of the resultant harvested crop and quality of the harvested crop, rather than just yield. This will require additional experimental studies to be performed and is beyond the scope of the current paper.

## 7 Conclusion

Commercial mushroom cultivation is a complex process, with many factors playing a role in determining yields, from compost composition and quality to environmental factors throughout the growing process. Thus far, guidance regarding determining environmental schedules (including e.g. temperature, humidity, oxygen and CO_2_ levels) has been relatively ad-hoc and has a limited quantitative basis. This is partly because of the difficulty in ascertaining the effect of these time-series variables on the growing process.

In this paper we applied the recently introduced Variable-Domain Functional Regression (VDFR) technique [12] to the domain of Agaricus Bisporus (button mushroom) yield modelling. This is particularly innovative, because it enabled us to understand the effect of the different functional variables on the yield, even when the growing process itself varied in length. We showed that a grower can obtain meaningful interpretations from the estimated coefficient functions, and we found that, in the case of the growing process used at Kyriakides Mushrooms Ltd., higher oxygen levels contribute negatively in the first two-thirds of the growing process, but they improve yields in the final third.

VDFR has previously only been used for modelling the condition of patients in a hospital, but we would argue that it would be particularly useful for the modelling of yields in crops that are, similar to mushrooms, grown in controlled conditions (e.g. tomatoes, strawberries etc.). While increasing yields is indeed vital for many growers, there are certainly extensions we are keen to consider, such as extending shelf-life and improving quality. This would be interesting from a statistical perspective also, as we can now consider how to extend VDFR to a multi-variate setting.

In concluding this section, we note that this research project undertaken with our industry partner was deemed a success from a practical perspective. Consequently, we are currently in the process of planning a larger scale project. The implementation of our framework in the commercial setting has been partially adopted by the industry partner so far, they have indicated that this has indeed improved performance and yield of their crops. In the follow up study we are aiming to take into account further details in addition to the yield considerations which include: quality ratings of the crop realized, early phased harvesting, and size/weight of each individual unit. In practice, from a commercial perspective, these are important considerations to make when selling such products in the market.

We note that when scaling up this study, we would also like to consider further the influence of the fertilizer type and its constituent attributes, which include the rate at which the soil is defrosted from its frozen state, when delivered. Furthermore, the thickness of the soil cover is deemed to be important to the growing surface area. We wish to consider these aspects in scaled up studies to determine the influence such features may have on the yield and crop quality.

The original data utilised to perform this anlaysis is available in the supporting information file.

## Supporting information

S1 Data This is the data used for the project. (ZIP)
