# Correction: Statistical modelling for precision agriculture: A case study in optimal environmental schedules for Agaricus Bisporus production via variable domain functional regression

**DOI:** 10.1371/journal.pone.0280374

**Published:** 2023-01-06

**Authors:** Efstathios Panayi, Gareth W. Peters, George Kyriakides

## Notice of republication

This article was republished on December 29^th^, 2022, to address a file issue. Please download this article again to view the correct version.
